# Fighting Through the Heat: How Male Aggression Influences Demography Under Recurrent Heatwaves

**DOI:** 10.1002/ece3.72034

**Published:** 2025-09-18

**Authors:** Neelam Porwal, Jonathan M. Parrett, Agnieszka Szubert‐Kruszyńska, Neha Pandey, Robert J. Knell, Tom C. Cameron, Jacek Radwan

**Affiliations:** ^1^ Evolutionary Biology Group, Faculty of Biology Adam Mickiewicz University Poznań Poland; ^2^ School of Natural Sciences University of Hull Hull UK; ^3^ School of Life Sciences University of Essex Colchester UK

**Keywords:** alternative mating tactics, demography, experimental evolution, extinction, heat, population bottleneck, small populations, stress

## Abstract

Sexual selection is a potent evolutionary force that can enhance adaptation and reduce mutational load, while simultaneously reducing survival, or causing sexual conflict that reduces fitness of one or both sexes. Many populations face extreme, short‐term stress events like heatwaves. The combined effects of sexual and environmental selection on population demography during and after such events remain poorly understood, even though such combined effects could be crucial for the persistence of small, endangered populations under climate change. In this study, we investigated how male aggression affects survival in a population during environmental stress. This was done by manipulating the expression of an aggressive male fighter morph in small populations of the male‐dimorphic mite *Sancassania berlesei,* using pheromonal cues from high‐density populations. We then exposed some of these populations to recurrent periods of extreme heat and monitored survival over eight generations. We found that heat exposure reduced survival, more severely in females than in males, and survival was lower in populations with higher fighter prevalence, but there was no interaction between temperature and fighter prevalence. Furthermore, survival declined across generations, and the decline was steeper in populations with lower prevalence of fighters, leading to the loss of their initial survival advantage by the last generation. Three populations exposed to heat went extinct from the reduced fighter expression regime. Our findings imply that despite its cost to individual survival, male aggression does not exacerbate population sensitivity to heatwaves over generations. Furthermore, we demonstrate that the mortality costs of male aggression are gradually compensated over successive generations, which could be a result of a more effective purging of inbreeding depression. Thus, while the additive effect of aggression and heatwaves on survival may increase demographic risks for bottlenecked populations in the short term, sexual selection may increase the resilience of populations to prolonged bottlenecks.

## Graphical abstract

This study examines how male aggression influences population survival under recurrent heat stress using experimental evolution in *Sancassania berlesei*. We show that while sexually selected tr initially reduce individual survival, they do not increase sensitivity to heatwaves and may enhance long‐term resilience to bottlenecks possibly by facilitating the purging of inbreeding depression.

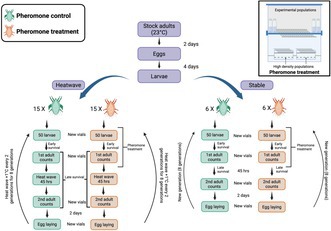

## 1 Introduction

Global environmental change, including rising temperatures, present substantial risks to biodiversity (Habibullah et al. 2022; Ripple et al. 2017), with notable declines forecast for species lacking adaptive capacity or dispersal abilities even with a modest increase of 1°C–2°C in temperature (Nunez et al. 2019). One of the most important predicted consequences of climate change is increased variability of climate, and a crucial aspect of this is likely to be an increase in the frequency and the amplitude of heatwaves. Heatwaves are known to have stronger impacts on life than directional changes (Sheldon and Dillon 2016) by directly influencing individual survival, physiological processes, and reproductive efficacy (Boni 2019) with these effects being more pronounced in ectothermic species (Kingsolver et al. 2013; Sen Ma et al. 2021; Neven 2000; Walsh et al. 2019). Under such conditions populations may be selected to evolve towards enhanced thermal tolerance, with traits that may enhance survival in high‐temperature environments being favoured, potentially allowing “evolutionary rescue” to occur (Bell 2017). However, adaptation may be severely limited when populations decline to the size that limits the amount of genetic variance available for selection (Frankham 2010). Furthermore, small population sizes result in significant genetic drift and inbreeding depression (Spielman et al. 2004). This, when combined with environmental stressors, can create a harmful cycle referred to as the “vortex of extinction” that can elevate the risk of extinction (Godwin et al. 2020; Ivimey‐Cook et al. 2021; Reed et al. 2012; Soulé et al. 1986). Understanding how populations adapt before environmental change drives them to extinction is a key research focus (Hoffmann and Flatt 2022; McCulloch and Waters 2023; Nunney 2016). One mechanism that may influence adaptability is sexual selection with multiple processes identified that drive the co‐evolution of thermal ecology and sexually selected traits. Key processes such as mortality due to male aggression, sex specific thermal sensitivity and consequences of sexual conflict on mortality and fecundity illuminate the role of sexual selection in facilitating thermal adaptation. Anthropogenic warming affects sexual selection, which can either promote adaptation by eliminating harmful alleles or hinder it through inbreeding and the evolution of costly traits (Leith et al. 2022). As a result, temperature stress can alter sexual selection dynamics, potentially impacting population persistence (García‐Roa et al. 2020).

Sexual selection is a process arising from competition for mates and their gametes that favours traits which bestow competitive advantages in the context of mating. For example, males may possess intricate displays serving to attract females or armaments and behaviours useful in direct competition for them or seminal fluids aiding competition for gametes (Andersson 1994; Darwin 1871). Additionally, traits used in reproductive competition are costly and therefore only the males in good condition, reflecting their adequate adaptation and/or low mutation load, should achieve higher reproductive success (Zahavi 1975; Rowe and Houle 1996). This could align sexual selection with natural selection, resulting in positive effects of sexual selection on population viability (Candolin and Heuschele 2008; Lorch et al. 2003).

Sexually selected traits may, however, decrease survival of their bearers (Ditchkoff et al. 2001; Endler 1980), or cause sexual conflict via their negative side effects on female fitness, directly, e.g., when coercive copulations inflict harm on females (Parker 1979; Rowe et al. 1994) or indirectly, via negative pleiotropic effect on female fitness (Harano et al. 2010; Rice and Chippindale 2001), potentially leading to net negative effects on population viability (Flintham et al. 2023). Strong skew in reproductive success resulting from sexual competition or skew in sex ratio due to male harm on females may further amplify these negative consequences by reducing effective population size (Grayson et al. 2014), increasing drift and inbreeding at the cost of adaptability. All these possibilities imply that sexual selection may be of key significance for populations endangered with extinction, particularly under environmental challenge (Kokko and Brooks 2003). The costs of exaggerated sexually selected traits and associated sexual conflicts (García‐Roa et al. 2019; Parsons 1995; Riechert 1988), combined with the negative consequences for survival of environmentally challenged populations discussed above, possibly are further augmented by demographic stochasticity (Martínez‐Ruiz and Knell 2017; Tschol et al. 2023). Sexual selection can influence both the immediate effects of environmental stress on individual fertility and survival (Baur et al. 2024; García‐Roa et al. 2020; Moiron et al. 2022), and long‐term adaptation (Gómez‐Llano et al. 2024; Iglesias‐Carrasco et al. 2024; Parrett and Knell 2018; Plesnar‐Bielak et al. 2012).

Studies on the effects of sexual selection on population viability so far have reported variable outcomes. Positive effects of sexual selection are well supported in experimental evolution studies (reviewed in Cally et al. 2019), with both purging of mutational load in stable environments (e.g., Lumley et al. 2015; Parrett et al. 2022) and enhanced adaptation to novel environments (e.g., Plesnar‐Bielak et al. 2012) being demonstrated. However, other sets of laboratory studies have reported significant negative effects of sexual selection via sexual conflict (Berger et al. 2016; Holland and Rice 1999). Additionally, comparative studies have linked elaboration of male sexually selected traits to increased risk of extinction (Bro‐Jørgensen 2014; Martins et al. 2018; but see Morrow and Fricke 2004; Morrow and Pitcher 2003), although other studies reported the reverse relationship in populations subjected to anthropogenic disturbances (Moore et al. 2024; Parrett et al. 2019), suggesting interactive effects of environmental factors.

Experimental evolution studies allowing manipulation of both sexual selection and environmental stress are well suited to examine such interactive effects. Most studies to date have manipulated sexual selection by either manipulating its intensity by altering adult sex ratios or manipulating mating systems either allowing polyandry or enforcing monogamy. Such manipulation during (Plesnar‐Bielak et al. 2012) or prior to (Berger and Liljestrand‐Rönn 2024; Godwin et al. 2020; Iglesias‐Carrasco et al. 2024) environmental challenge generally showed positive effects of sexual selection, particularly in environments limiting the possibility of female harassment by males (Berger and Liljestrand‐Rönn 2024; Yun et al. 2018). Most of these studies focused on step or gradual environmental change, whereas extreme weather anomalies, such as heat waves, may dominate environmental effects on demographics of natural populations, as discussed above. Furthermore, these studies mostly focus on consequences of manipulation of ecological processes (e.g., via sex ratio manipulation or by enforced monogamy), these manipulations can alter the demographic parameters we would be measuring on a population scale. Indeed, one study which did manipulate sexual selection by using evolutionary lines artificially selected for or against the presence of armoured aggressive males found contrasting results. In *Rhizoglyphus robini* exhibiting alternative reproductive phenotypes, lines nearly fixed for costly weapons were more likely to go extinct under gradual temperature increase (Łukasiewicz et al. 2023).

We conducted a multi‐generational experiment to examine how male morph ratios influence population demography under heatwaves using acarid mite *Sancassania berlesei*. This species exhibits similar male dimorphism to *R. robini*, but with morph heritability being low and mostly determined by population density during nymphal development (Radwan 1995). As the cue to the density is provided by pheromones (Radwan et al. 2002), we can suppress the expression of fighter morphs in populations independently of genetic background using pheromonal cues from high‐density populations. This allowed us to investigate the effect of manipulation of proportions of sexually selected males on population demographics in the context of thermal stress. We contrasted populations with a higher proportion of fighter males (pheromone control) against those with a lower proportion of fighter males (pheromone treatment). Within each pheromone regime, we either maintained populations under a stable temperature environment or subjected them to successive periods of extreme heat over eight generations to simulate the thermal stressors many species may encounter due to climate change. Importantly, this kind of intermittent stress may have only passing effects on individual condition, and thus not suppress the expression of costly sexually selected traits. This is a relevant point because, by reducing associated costs, such suppression by chronic stress (e.g., associated with permanent environmental change) was hypothesised to alleviate negative effects of sexual selection on population viability (Kokko and Brooks 2003). To ensure the expression of fighter morph is not suppressed by stress in our study, we applied it at the adult stage when the morphs are fixed.

We predicted that a high prevalence of fighter males will have a negative effect on the survival of males due to fights, but possibly also of females that are also sometimes killed by fighter males (Łukasik 2010). We further predicted that heatwaves (defined as 45 h of extreme rise in temperatures in our experiment) would increase mortality and may have disproportionately more detrimental effects on fighters because their expression of costly weapons could make them less tolerant of additional costs imposed by thermal stress.

## 2 Methods

### 2.1 Housing and Maintenance of Mite Populations

For this study, we used soil mites (*S. berlesei*), originally collected from poultry litter from a farm in Dluga Goslina, Wielkopolska region of Poland, in 2022. These populations have since been maintained under controlled conditions, with overlapping generations and large population sizes (> 1000 adults). Populations were housed in two custom‐designed bottle‐top enclosures consisting of the bottleneck and cap with a 5 mm hole tightly sealed with a cotton plug, and a cemented base to balance ventilation and containment. Maintenance conditions included high humidity levels (over 90%) and a constant temperature of 23°C in dark incubators. The mites were fed dry yeast twice weekly and were transferred to new bottle‐tops and mixed between two bottle‐tops once monthly.

### 2.2 Experimental Populations

To obtain generation‐synchronised experimental populations, adult mites from the stock populations were isolated into a new box for a 2‐day egg‐laying period. After the eggs hatched (4 days after adults were removed), 50 larvae were transferred to each of 42 glass vials (2.5 cm × 5 cm). The bottom of each vial was covered with a 2.5 cm layer of plaster of Paris to create a water‐absorbent substrate. The vial lids had a 10 mm hole covered with fine mesh to facilitate air exchange while preventing mite escape. To minimise disturbance, excess food was provided but only during vial transfers. Populations were assigned to one of two pheromone regimes: pheromone treatment was used as described below to decrease the proportion of fighters using pheromones in a population compared to pheromone control populations that were not exposed to pheromones. These were further divided into temperature (heatwave vs. stable). It included 15 pheromone‐treated and 15 pheromone control populations under heatwave conditions, and 6 pheromone‐treated and 6 pheromone control populations under stable conditions. More heatwave populations were tested because heat was expected to cause extinctions, needing more replicates to ensure sufficient data.

### 2.3 Pheromone Treatment

**FIGURE 1:**
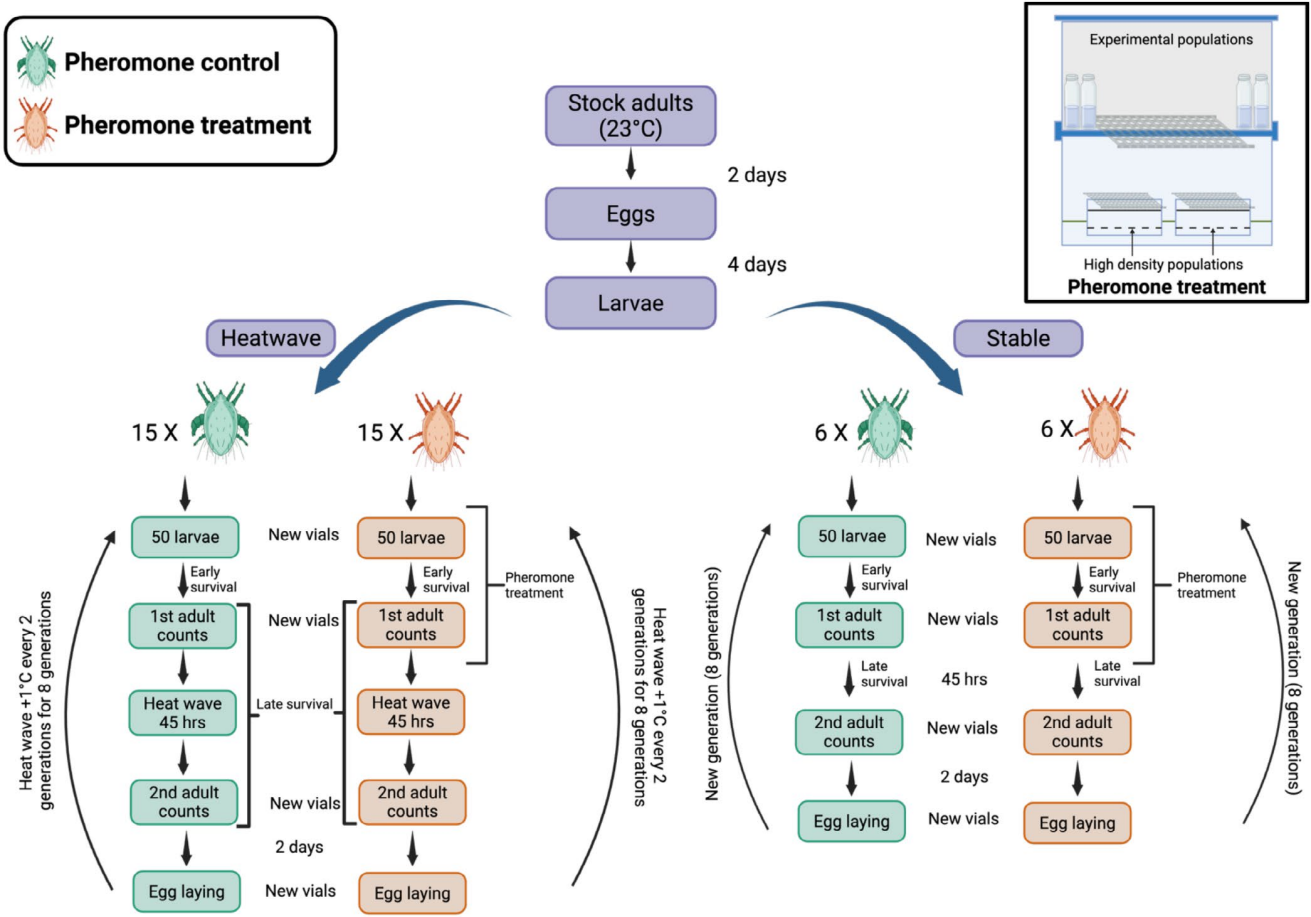
Schematic of the experimental workflow, illustrating the pheromone control (green) and pheromone treatment (orange) regimes. Stock adults (23°C) produced eggs that developed into larvae over 4 days before being assigned to either heatwave or stable environments. Pheromone treatment populations were exposed to high‐density pheromone cues from the larval stage until the first adult counts in the setup on the top right. Key experimental steps, including survival assessments, developmental stages, and reproductive phases, are outlined for both conditions. Created in https://BioRender.com.

Populations from both pheromone treatment regimes, during their development from larvae to adults (pheromonal sensitive window of morph determination), were maintained in the pheromone treatment setup. This setup consisted of two transparent plastic boxes; one placed atop the other, separated by a lid. The lid of the bottom box and the base of the top box had square cutouts covered with mesh to allow pheromone diffusion between the boxes. Bottom boxes contained two high‐density mite populations housed in plastic containers. Each container had a 1 cm cement base and was filled with a ~2 cm layer comprising a 1:1 ratio of food to mites. While these populations were not counted, we estimate according to the size of the container that each contained more than one hundred thousand mites. The containers were surrounded by dishwashing liquid to prevent mites from contaminating experimental populations. The populations from the pheromone treatment populations were placed in the top box, which was covered with a lid featuring holes covered with porous tape to permit restricted air exchange (Figure 1). Concurrently, both pheromone control regimes were maintained in an identical setup without the high‐density mite populations to ensure consistent environmental conditions. Logistically, it was only possible to set up one container each for pheromone control and pheromone treatment and then transfer to different temperature treatments respectively, but the containers hosting both treatments were identical except for the presence of dense colonies in pheromone treatment. Hence, we do not expect any uncontrolled difference between containers to affect our results.

### 2.4 Experimental Workflow

Larvae from the F_0_ generation were transferred into 42 vials and randomly assigned to one of two treatment groups: pheromone exposure (treatment) or no pheromone exposure (control). Vials were maintained at 23°C, and treatments were applied for a period of 6–8 days. First adult counts were conducted 6 days post larvae transfer, one day beyond the minimum development time at this temperature to develop into adults. This was done to maximise the proportion of emerging adults (> 85% in all but four vials) while minimising fight‐related mortality. This timing ensured sufficient maturation across the majority of individuals prior to assessment. Tritonymphs were also counted in the first adult counts for each generation. These first adult counts were taken while transferring mites into new vials. Thereafter, heatwave populations were subjected to a heatwave for 45 h. The heatwave temperature initially started at 34°C (F_0_) and was increased by 1°C every two generations, reaching 38°C by the final generation. The temperature range was chosen from the pilots to get 20%–50% female mortality. Temperatures were increased by a degree every generation to explore the gradual increase in temperature for a short‐term heatwave every generation to be expected under current environmental crises. Second adult counts were conducted while simultaneously transferring mites to new vials for a 2‐day mating/egg‐laying period. Adult mites were sexed for both counts, and fighter males were distinguished from scramblers. Subsequently, the adults were killed, and the eggs were given 4 days to hatch. For the next generation, 50 larvae were typically transferred. In a few cases (3.17%), first adult counts slightly exceeded 50 (by 1–3 adults), and the number of larvae was changed to the number of adults in such cases to avoid exclusion. If fewer than 50 larvae were available, nymphs were included to reach a total of 50 individuals or as close as possible. This protocol was repeated for each generation (Figure 1).

A population was considered extinct if (a) there were no larvae or (b) there were either no adults or no females in the first count.

### 2.5 Statistics

Survival data were initially analysed using generalised linear mixed effects models (GLMM) with binomial error structures. If diagnostics indicated issues with over‐dispersion, GLMMs with a beta‐binomial error distribution were used. The number of females (count data, Gaussian distribution) was analysed using a linear mixed effects model (LMM). The glmmTMB package (Brooks et al. 2017) was used for fitting all the models, and diagnostic plots were generated using the DHARMa package (Brooks et al. 2024; Hartig 2024). Statistical models included fixed effects of pheromone, temperature, and generation (continuous), with two‐way and three‐way interactions with generations. The Pheromone × Temperature interaction shows how effects of fighter prevalence vary with heat. Pheromone × Generation and Temperature × Generation interactions indicate how these effects evolve over generations, reflecting adaptation or responses to rising temperatures. The three‐way interaction assesses if combined pheromone‐temperature effects shift across generations, revealing dynamic population responses. Population ID was entered as a random effect. Each model with a full set of explanatory variables and their interactions was reduced to minimal adequate models in which only significant explanatory variables were retained (Crawley 2012). All analyses were performed in R version 4.4.1 (R Core Team 2024).

To estimate the effectiveness of our pheromone treatment, the proportion of fighter morph in each count was analyzed as a binary response variable, e.g., cbind(fighters, scamblers). For this, we used the subset of populations for which all juveniles reached adulthood at the first count, as it was not possible to determine the morph of the tritonymphs. This limited our sample size to 68.3% of the total dataset. Count (first or second adult counts) was entered as an additional fixed effect.

For analysing the proportion of individuals surviving at each generation, we used a binary response variable in the form of a vector containing the numbers of survived and dead individuals. The proportion survival inferred from two subsequent counts within a population, i.e., dead = the number initially present—number survived. It was analysed separately for juveniles to first adult counts (early survival) and first to second adult counts (late survival). F_0_ counts were not included in analyses of early survival because this stage was not exposed to direct or cross‐generation effects of heatwaves. For the sex and morph specific survival, we used a subset of populations without tritonymph, same as in the analysis of fighter proportions, but with sex and morph as a fixed effect respectively instead of count. We further analysed the proportion of females in the population only for the second adult counts to additionally see the indirect effects of fight related male mortality on adult sex ratios. For this, we used the proportion of females as a binary response variable. Furthermore, we analysed the absolute counts of females from the second count, as this number sets an upper limit on the productivity of the population for the next generation.

## 3 Results

None of the populations in the stable temperatures went extinct. No population went extinct in control treatment (high fighter prevalence), but three (*N* = 3/15) of the pheromone treatment (lower fighter prevalence) heatwave populations went extinct in the second, fourth, and the sixth generation, respectively. Two of the populations became extinct because there were no females in the first adult counts, while the third one became extinct due to no adults (only one tritonymph) in the first adult counts. Additionally, for most of the populations, we had 50 larvae to start the next generations, except for 5.9% of the time in pheromone treatment with heatwaves.

### 3.1 Fighter Proportions

**FIGURE 2:**
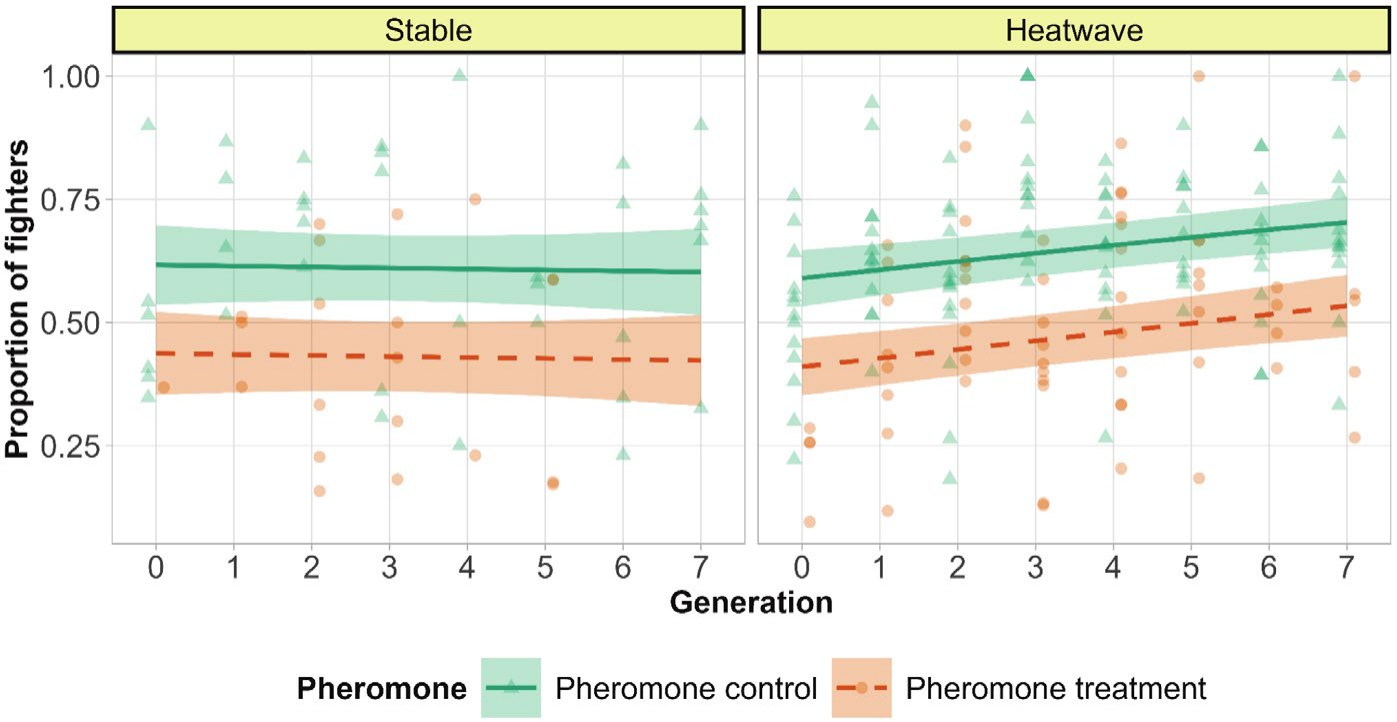
Fighter proportion in the stable and heatwave regimes. Count was not retained in the final model, so for graphical representation of the model, the average proportion of fighters from the two counts was presented. The lines are predicted by the model; points are raw data. They denote pheromone control (higher fighter prevalence) and orange dashes and circles denote pheromone treatment (lower fighter prevalence) with smooth areas as 95% confidence intervals.

Pheromone exposure caused a reduction in the proportion of males developing into the fighter morph: the proportion of fighter males in the pheromone control was significantly higher than in the pheromone treatment, with ~64% of males being fighters in pheromone control and ~40% in pheromone treatment across the entire experiment (main effect of pheromone treatment: *χ* ^2^ = 21.524, df = 1, *p* < 0.001, Figure 2, Tables S1 and S2), confirming the effectiveness of pheromone treatment. Additionally, there was a significant temperature × generation interaction indicating that the proportion of fighters increased over time in the heatwave treatments only (*χ* ^2^ = 4.724, df = 1, *p* = 0.030, Figure 2, Tables S1 and S2).

### 3.2 Survival

**FIGURE 3:**
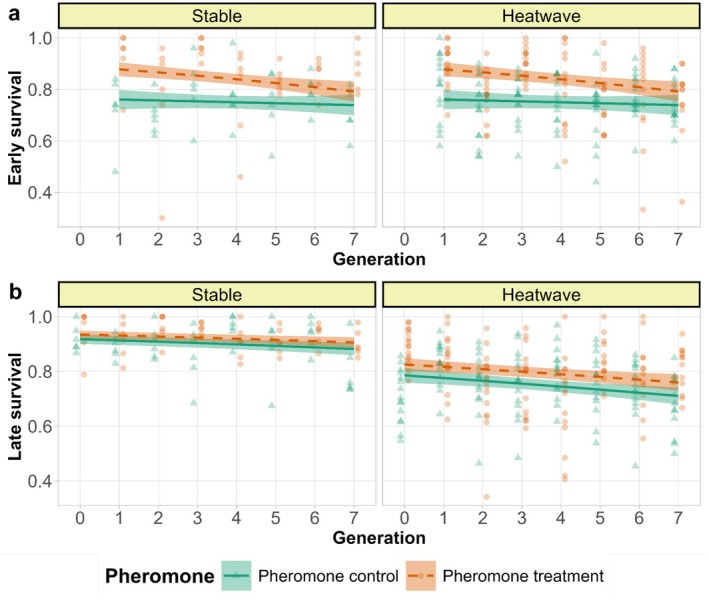
Proportion of survived individuals over generations in two temperature regimes indicated in the panels. (a) Early survival, (b) Late survival over generations in temperature regimes in the panels. The lines are predicted by the model and points are raw data. The green lines and triangles denote pheromone control (higher fighter prevalence) and orange dashes and circles denote pheromone treatment (lower fighter prevalence). The smooth areas are 95% confidence intervals. Temperature regimes are indicated on the panels.

Late survival was higher in pheromone treatment than in pheromone control (*χ* ^2^ = 10.115, df = 1, *p* = 0.002, Tables S3 and S4) and late survival was reduced by exposure to heatwaves (*χ* ^2^ = 70.897, df = 1, *p* < 0.001, Figure 3b, Tables S3 and S4). Survival declined over generations in all treatments (*χ* ^2^ = 11.774, df = 1, *p* < 0.001, late survival, Figure 3, Tables S3 and S4). However, the decline in early survival for the pheromone treatment over generations was higher than for the pheromone control, as indicated by a significant pheromone × generation interaction, such that the initial higher survival in pheromone treatment populations largely disappeared towards the last generation (*χ* ^2^ = 4.208, df = 1, *p* = 0.040, Figure 3a, Tables S3 and S4). Additionally, none of the three‐way interactions were significant.

**FIGURE 4:**
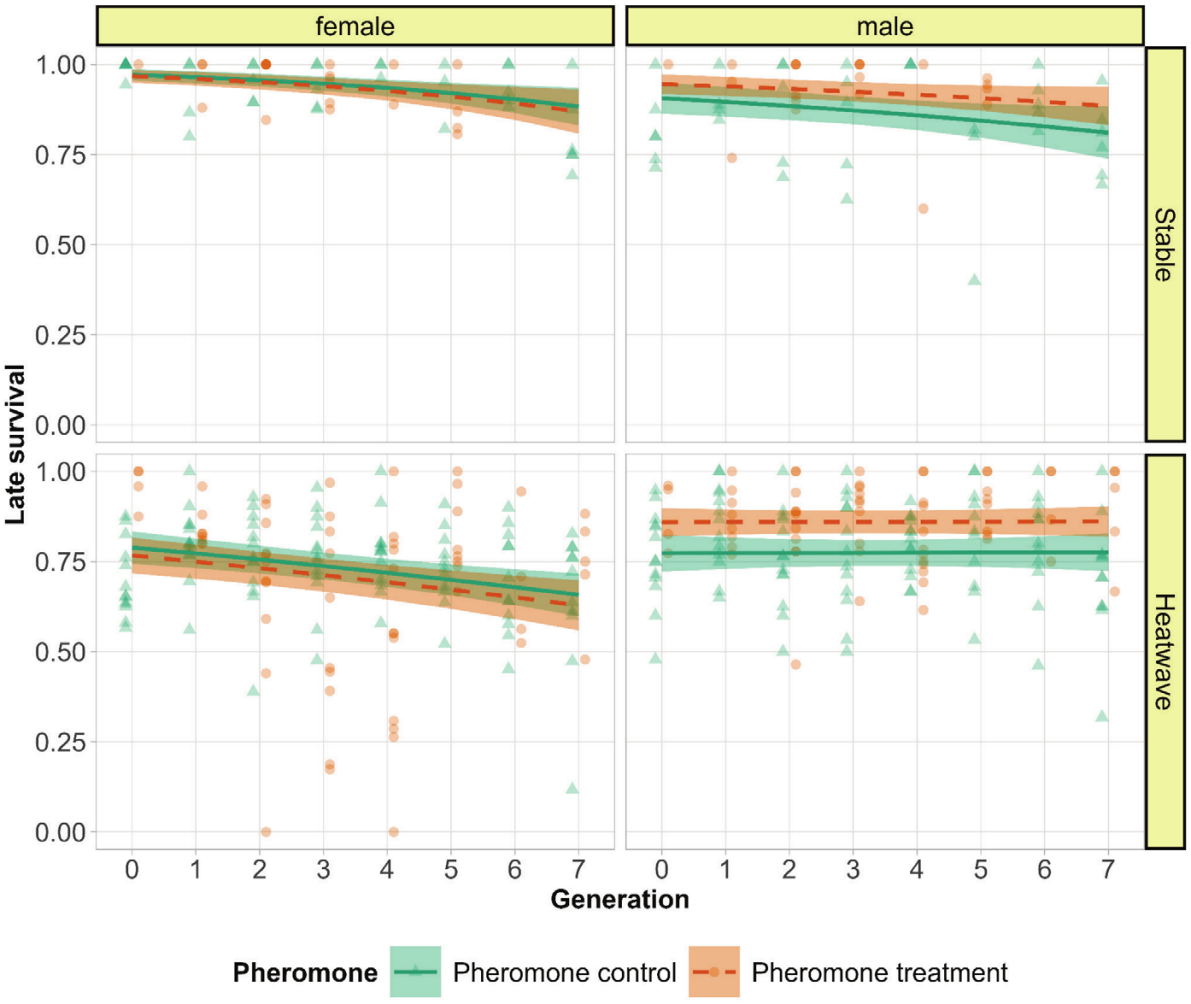
Sex‐specific late survival. The lines are predicted by the model and points are raw data. The green lines and triangles denote pheromone control (higher fighter prevalence) and orange dashes and circles denote pheromone treatment (lower fighter prevalence) with smooth areas as 95% confidence intervals. The panels are sex in vertical and temperature regimes in horizontal.

**FIGURE 5:**
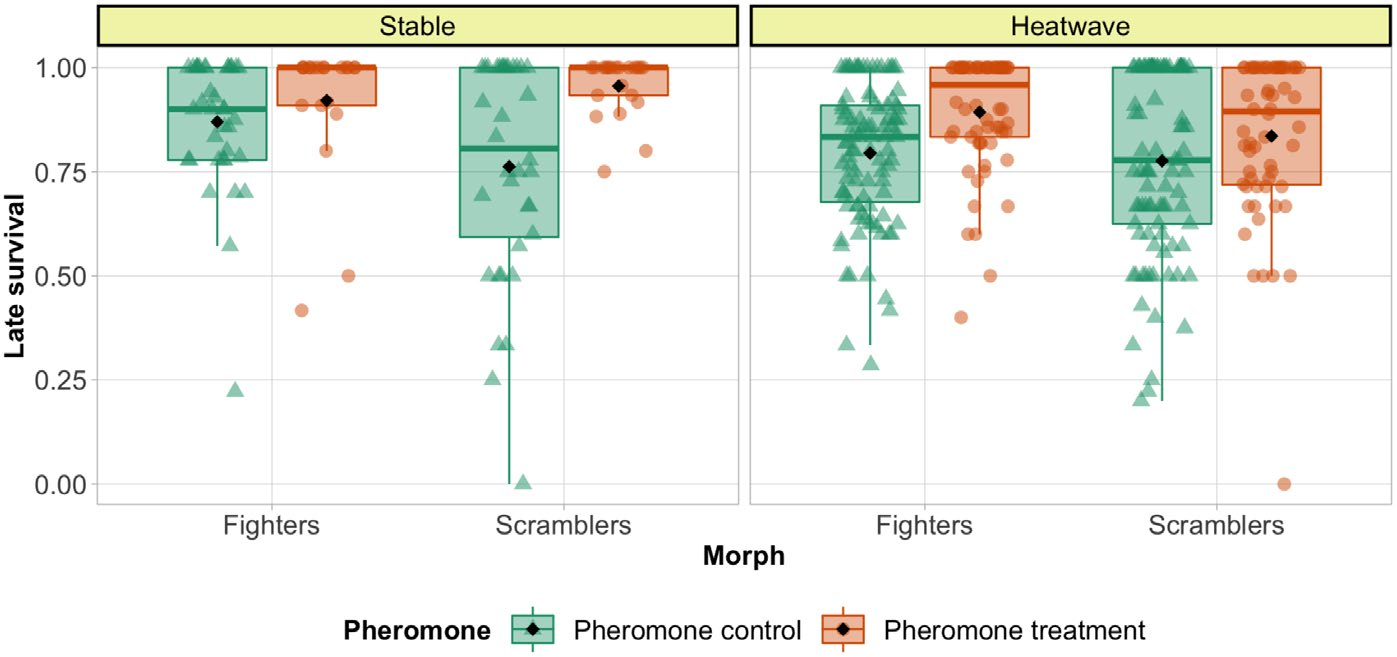
Morph specific late survival. The green boxes and triangles denote pheromone control (higher fighter prevalence) and orange boxes and circles denote pheromone treatment (lower fighter prevalence) and the panels are temperature regimes. The box shows dispersion of data in medium interquartile range (25%–75% of data) while the line in box is the median, and whiskers show 1.5 × interquartile range. Black diamonds are the means.

Females were more sensitive to heat than males, indicated by a significant two‐way interaction between temperature and sex (*χ* ^2^ = 23.957, df = 1, *p* < 0.001, Figure 4, Tables S5 and S6). Sex also interacted with pheromone treatment, with males, but not females, from pheromone treatment lines initially surviving better than in pheromone control lines (*χ* ^2^ = 15.742, df = 1, *p* < 0.001, Figure 4, Tables S5 and S6). Furthermore, a significant sex–generation interaction indicates that female survival declined more than male survival over generations (*χ* ^2^ = 6.187, df = 1, *p* = 0.013, Figure 4, Tables S5 and S6). Additionally, none of the three‐way interactions were significant. Fighters in general survived better than scramblers (*χ* ^2^ = 4.311, df = 1, *p* = 0.038, Figure 5, Tables S7 and S8).

### 3.3 Proportion and Number of Females at the Onset of Reproduction for Larvae Collection

**FIGURE 6:**
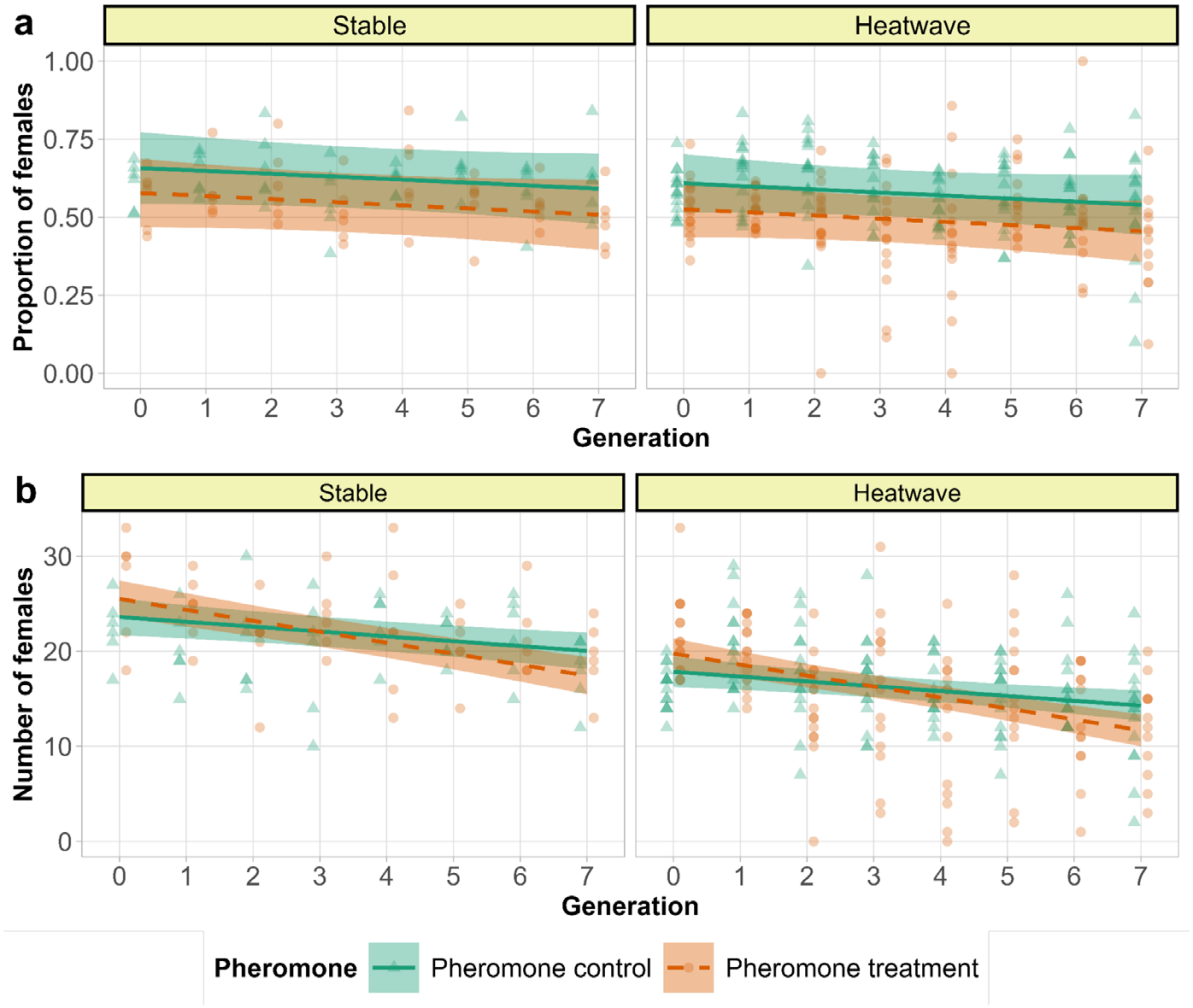
(a) Proportion of females and (b) number of females in the second counts. The lines are predicted by the model and points are raw data. The green lines and triangles denote pheromone control (higher fighter prevalence) and orange dashes and circles denote pheromone treatment (lower fighter prevalence) with smooth areas as 95% confidence intervals. Temperature regimes are indicated on the panels.

Pheromone control populations were more female biased than the pheromone treatment (*χ* ^2^ = 33.856, df = 1, *p* < 0.001, Figure 6, Tables S9 and S10). The number of females at second adult counts was negatively affected by heatwave regime (*F* = 33.678, df = 1, *p* < 0.001, Figure 6, Tables S9 and S10). Furthermore, there was a significant pheromone–generation interaction, with the decline in the number of females over generation being steeper in pheromone treatment than in pheromone control (*F* = 7.456, df = 1, *p* = 0.007, Figure 6, Tables S9 and S10).

## 4 Discussion

The demographic consequences of strong sexual selection, particularly in small populations vulnerable to inbreeding and reduced adaptive capacity, are only beginning to be explored. In this study, we manipulated the prevalence of sexually selected males by suppressing the expression of armed, aggressive fighter morphs and examined the effects of heatwaves on survival. As predicted, both higher sexually selected trait expression and heatwaves reduced survival. These effects were sex‐specific, with sexually selected trait expression primarily affecting males, while heatwaves had a stronger impact on females. We found no support, however, for our prediction that fighter males would experience disproportionately greater mortality under heatwaves because of the costs associated with weapon expression. We additionally found that survival declined over generations, independently of the direct effects of heatwaves. We discuss these findings and their broader implications in detail below.

Experimental heatwaves reduced late survival compared to controls. Sex‐specific analysis showed that females were more sensitive to heatwaves than males. Heatwaves reduced the number of reproductive females, a key factor in population dynamics. While for logistic reasons we were not able to measure female fecundity, females lay eggs continuously at a relatively constant rate for the first few days of life, with a peak fecundity at days 3–4 (Radwan 1993); therefore, survival during this period is a good proxy for fitness. A review by Edmands (2021) found no consistent sex‐based differences in heat tolerance, but higher female resilience was more common. Although these results contrast from a closely related species *R. robini*, where no sex differences in survival during heatwaves were reported (Parrett et al. 2024). However, we do not currently understand the mechanism behind these differences. Independently, pheromone treatment affected male survival, with reduced survival in pheromone control populations where fighter morphs were more prevalent. This was accompanied by more female‐biased sex ratios, suggesting elevated male mortality, likely due to lethal male fights (Radwan 1993). Contrary to our predictions, heat and pheromone treatments did not significantly interact in their effect on late survival. Inconsistent with the proposition that increased male mortality associated with the expression of costly sexually selected weapons may be aggravated when conditions are poor (Bro‐Jørgensen 2014; Kokko and Brooks 2003; Martínez‐Ruiz and Knell 2017), we found no differences between male morphs in their sensitivity to heat stress, consistent with the results in *R. robini* (Parrett et al. 2024). Furthermore, despite significantly female‐biased sex ratios in higher fighter‐prevalence populations, we noted no cases when there were no males at the mating/oviposition period. Additionally, we did not observe any extinctions in pheromone control heatwave populations, despite increased male mortality, even though we did note three extinctions in lower fighter‐prevalence heatwave populations. Thus, despite associated reduced mortality in the pheromone treatment population, decreased prevalence of sexually selected males did not result in decreased male susceptibility to heatwaves. It is possible that fighter phenotypes are better buffered against desiccation due to their investment in more sclerotised cuticle, which could compensate for the increased cost of sexually selected phenotype under heat stress (Łukasiewicz 2020).

Beyond the effects of heat and pheromone treatment, early and late survival declined over generations. Given that survival is among the traits most sensitive to inbreeding across taxa (DeRose and Roff 1999), this generational decline could be due to the negative impact of the bottleneck of a maximum of 50 individuals imposed by our design over 8 generations (Frankham 2010; Spielman et al. 2004). Assuming an N/Ne ratio of the order of 0.25 (Palstra and Fraser 2012), this would have resulted in a cumulative inbreeding coefficient of (1‐(1–1/Ne)^8^ = 0.28), i.e., the order of one generation of sib‐mating (Frankham 2010). The generational decline in survival was more pronounced in females, as indicated by a significant two‐way interaction. Environmental challenges may exacerbate inbreeding effects (Reed et al. 2012), for which we found mixed support. While there was no significant temperature*generation interaction for early survival (Tables S3 and S4), we observed one for late survival, but only in a model accounting for sex (Tables S5 and S6). This may be because heatwave effects were sex‐specific, and ignoring this reduced statistical power by inflating within‐population variance in survival.

Generational declines in survival could possibly be mediated by sexual selection via reduced effective population sizes and increased range of inbreeding associated with increased male mortality. However, the decline was not steeper in pheromone control populations, nor did it interact with heat. In contrast, early survival declined more steeply in pheromone treatment populations, eroding their initial survival advantage by the last generation (Figure 3a, Tables S3 and S4). A similar interaction between pheromone treatment and generation was also observed for the number of females surviving till experimental egg‐laying, which was initially lower in pheromone control treatment but with the rank reversed at the end of the experiment (Figure 6). These results demonstrate that long‐term positive effects of sexual selection on population dynamics in small populations can at least compensate for the negative effects of higher mortality it induces. The compensation is likely due to stronger selection against recessive deleterious alleles revealed by progressing inbreeding (Charlesworth and Willis 2009). Indeed, enhanced purging of inbreeding load via sexual selection manipulated by enforcing monogamy have been demonstrated in studies in another acarid mite *R. robini* (Jarzebowska and Radwan 2009) and in flour beetles *Tribolium castaneum* (Lumley et al. 2015). Our data also suggest that increasing the prevalence of a sexually selected weapon in populations may have a similar effect and corroborate the findings of fighter selected populations purging genetic load in *R. robini* (Parrett et al. 2022).

Our results contrast with those obtained in another male‐dimorphic acarid, *R. robini* (Łukasiewicz et al. 2023), which showed that fighter‐selected populations exhibit poorer survival and greater extinction risk with rising temperatures. Here we observed only a few extinctions, but only in the heatwave regime with suppressed fighter expression. Additionally, irrespective of the heatwave regime, the initial survival advantage of these populations was decreasing over consecutive generations. One possible explanation for the contrasting conclusions is that the studies differed in the way heat stress was applied (heatwaves in the present study versus milder but permanent increase in Łukasiewicz et al. 2023). Another possible reason is the difference between the two species; they differ in the mode of male morph determination, being highly heritable in *R. robini* (Parrett et al. 2022; Radwan 1995) but predominantly environmentally cued in *S. berlesei* (Michalczyk et al. 2018; Radwan 1995). The effects of the genetic architecture of sexually selected traits and the way the heat stress is applied deserve to be explored by future studies.

To conclude, our study shows that sexual selection can influence the survival of small populations. While high fighter prevalence increased male mortality and initially reduced the number of reproducing females, it also enhanced resilience to prolonged bottlenecks, likely through purging of inbreeding load. However, our results do not support the hypothesis that sexual selection increases vulnerability to environmental challenges. Contrary to theoretical models (Kokko and Brooks 2003; Martínez‐Ruiz and Knell 2017), the negative effects of costly sexually selected traits were not exacerbated by environmental stress, nor did heatwaves interact with prolonged bottlenecks. We cannot, of course, rule out that increased male mortality from fights, combined with heatwave‐driven female losses, would ultimately reduce long‐term survival in small populations under strong sexual selection and demographic stochasticity (Martínez‐Ruiz and Knell 2017), although we only observed extinctions when fighter prevalence was low, indicating that this risk might be smaller than previously thought.

## Author Contributions

**Neelam Porwal:** conceptualization (equal), data curation (lead), formal analysis (equal), investigation (equal), methodology (equal), visualization (lead), writing – original draft (lead), writing – review and editing (lead). **Jonathan M. Parrett:** conceptualization (equal), formal analysis (equal), investigation (equal), methodology (equal), validation (equal), visualization (supporting), writing – review and editing (equal). **Agnieszka Szubert‐Kruszyńska:** investigation (equal), methodology (supporting). **Neha Pandey:** investigation (equal), writing – review and editing (supporting). **Robert J. Knell:** conceptualization (equal), formal analysis (supporting), funding acquisition (equal), methodology (supporting), project administration (equal), supervision (equal), validation (equal), visualization (supporting), writing – review and editing (equal). **Tom C. Cameron:** conceptualization (equal), formal analysis (supporting), funding acquisition (equal), methodology (supporting), project administration (equal), supervision (equal), validation (equal), writing – review and editing (equal). **Jacek Radwan:** conceptualization (equal), formal analysis (equal), funding acquisition (equal), methodology (equal), project administration (equal), supervision (equal), validation (equal), writing – review and editing (equal).

## Conflicts of Interest

The authors declare no conflicts of interest.

## Supporting information


**Tables S1‐S10.** ece372034‐sup‐0001‐TablesS1‐S10.docx.

## Data Availability

The dataset is available in the Dryad repository. Dataset DOI: https://doi.org/10.5061/dryad.1jwstqk6h
