# Supplementary material for: Fighting Through the Heat: How Male Aggression Influences Demography Under Recurrent Heatwaves
Source: Ecol Evol. 2025 Sep 18;15(9):e72034. doi: 10.1002/ece3.72034 (PMC12446712; doi:10.1002/ece3.72034)
Supplement: Supplementary file 1 — Tables S1‐S10. ece372034‐sup‐0001‐TablesS1‐S10.docx. [file ECE3-15-e72034-s001.docx]

**Fighting Through the Heat: How Male Aggression Influences Population Demography Under Recurrent Heatwaves**

**Supplementary Material**

**Table 1: Results from GLMM with betabinomial error structure to predict fighter proportions without including the first and second adult counts where first adult counts had tritonymphs.**
*The factors and interactions retained in the final model are denoted in bold. χ² values, d.f., and p-values are from likelihood ratio tests comparing models with and without the variable in question.*

| **Source of variation** | **χ²** | **d.f.** | **p-value** |
| --- | --- | --- | --- |
| temperature | - | - | - |
| **pheromone** | **21.524** | **1** | **<0.001** |
| generation | - | - | - |
| **temperature * generation** | **4.724** | **1** | **0.030** |
| count | 0.338 | 1 | 0.561 |
| pheromone * count | 0.035 | 1 | 0.852 |
| temperature * pheromone | 0.034 | 1 | 0.853 |
| count * generation | 0.018 | 1 | 0.894 |
| pheromone * generation | 0.008 | 1 | 0.930 |
| temperature * count | 0.003 | 1 | 0.956 |
| Temperature:Pheromone:generation | 0.418 | 1 | 0.518 |
| Temperature*Pheromone*count | 0.217 | 1 | 0.642 |
| Temperature*count*generation | 0.134 | 1 | 0.715 |
| Pheromone*count*generation | 0.039 | 1 | 0.843 |

**Table 2: Summary results from the model for fighter proportion**

| **Explanatory variable** | **est.** | **s.e.** | **z-value** | **p-value** |
| --- | --- | --- | --- | --- |
| Intercept | 0.476 | 0.174 | 2.737 | **0.006** |
| temperatureheatwave | -0.111 | 0.189 | -0.589 | 0.556 |
| pheromonetreatment | -0.727 | 0.136 | -5.356 | **<0.001** |
| generation | -0.008 | 0.031 | -0.267 | 0.789 |
| temperatureheatwave:generation | 0.080 | 0.037 | 2.180 | **0.029** |

**Table 3: Results from fitting GLMMs with betabinomial error structures to predict individual early and late survival.**
*The factors and interactions retained in the final model are denoted in bold. χ² values, d.f., and p-values are from likelihood ratio tests comparing models with and without the variable in question.*

| **Source of variation** | **Early Survival** | | | **Late Survival** | | |
| --- | --- | --- | --- | --- | --- | --- |
|  | χ² | d.f. | **p-value** | χ² | d.f. | **p-value** |
| temperature | 1.947 | 1 | 0.163 | **70.897** | **1** | **<0.001** |
| pheromone | - | - | - | **10.115** | **1** | **0.002** |
| generation | - | - | - | **11.774** | **1** | **<0.001** |
| **pheromone * generation** | **4.208** | **1** | **0.040** | 1.570 | 1 | 0.210 |
| temperature * pheromone | 3.433 | 1 | 0.064 | 0.490 | 1 | 0.484 |
| temperature * generation | 0.907 | 1 | 0.341 | 0.255 | 1 | 0.614 |
| Temperature*Pheromone*generation | 0.132 | 1 | 0.717 | 0.323 | 1 | 0.570 |

**Table 4: Model summary results from models for early and late survival**

| **Explanatory**  **variable** | **Early Survival** | | |  | **Late Survival** | | | |
| --- | --- | --- | --- | --- | --- | --- | --- | --- |
|  | est. | s.e. | z-value | p-value | est. | s.e. | z-value | p-value |
| Intercept | 1.176 | 0.127 | 9.264 | <0.001 | 2.413 | 0.117 | 22.554 | **<0.001** |
| temperatureheatwave |  |  |  |  | -1.113 | 0.100 | -11.106 | **<0.001** |
| pheromonetreatment | 0.904 | 0.200 | 4.516 | <0.001 | 0.253 | 0.077 | 3.301 | **<0.001** |
| generation | -0.019 | 0.027 | -0.716 | 0.474 | -0.057 | 0.017 | -3.453 | **<0.001** |
| pheromonetreatment:generation | -0.087 | 0.042 | -2.059 | 0.040 |  |  |  |  |

**Table 5: Results from fitting a GLMM with binomial error structure to predict sex-specific late survival without including the counts where first adult counts had tritonymphs.**
*The factors and interactions retained in the final model are denoted in bold. χ² values, d.f., and p-values are from likelihood ratio tests comparing models with and without the variable in question.*

| **Source of variation** | **χ²** | **d.f.** | **p-value** |
| --- | --- | --- | --- |
| temperature | - | - | - |
| pheromone | - | - | - |
| generation | - | - | - |
| sex | - | - | - |
| **temperature * sex** | **23.957** | **1** | **<0.001** |
| **temperature * generation** | **4.757** | **1** | **0.029** |
| **pheromone * sex** | **15.742** | **1** | **<0.001** |
| **sex * generation** | **6.187** | **1** | **0.013** |
| pheromone * generation | 1.377 | 1 | 0.241 |
| temperature * pheromone | 1.027 | 1 | 0.311 |
| temperature * sex * generation | 3.018 | 1 | 0.082 |
| pheromone*sex*generation | 1.760 | 1 | 0.185 |
| temperature*pheromone*generation | 0.907 | 1 | 0.341 |
| temperature*pheromone*sex | 0.238 | 1 | 0.625 |

**Table 6: Summary results from the model for sex-specific survival after heatwave.**

| **Explanatory variable** | **est.** | **s.e.** | **z-value** | **p-value** |
| --- | --- | --- | --- | --- |
| Intercept | 3.521 | 0.289 | 12.189 | **<0.001** |
| temperatureheatwave | -2.203 | 0.285 | -7.716 | **<0.001** |
| pheromonetreatment | -0.126 | 0.144 | 0.880 | 0.379 |
| sexmale | -1.248 | 0.271 | -4.602 | **<0.001** |
| generation | -0.214 | 0.055 | -3.873 | **<0.001** |
| temperatureheatwave:sexmale | 1.154 | 0.235 | 4.906 | **<0.001** |
| temperatureheatwave:generation | 0.119 | 0.055 | 2.174 | **<0.030** |
| pheromonetreatment:sexmale | 0.710 | 0.178 | 3.990 | **<0.001** |
| sexmale:generation | 0.097 | 0.040 | 2.491 | **<0.001** |

**Table 7: Results from fitting a GLMM to predict morph-specific late survival without including the counts where first adult counts had tritonymphs.**
*The factors and interactions retained in the final model are denoted in bold. χ² values, d.f., and p-values are from likelihood ratio tests comparing models with and without the variable in question.*

| **Source of variation** | **χ²** | **d.f.** | **p-value** |
| --- | --- | --- | --- |
| **temperature** | **12.507** | **1** | **<0.001** |
| **pheromone** | **16.856** | **1** | **<0.001** |
| **morph** | **4.311** | **1** | **0.038** |
| generation | 4.532 | 1 | 0.270 |
| temperature * pheromone | 2.480 | 1 | 0.115 |
| pheromone * generation | 0.634 | 1 | 0.426 |
| morph * generation | 0.662 | 1 | 0.416 |
| pheromone * morph | 0.130 | 1 | 0.718 |
| temperature * generation | 0.121 | 1 | 0.728 |
| temperature * morph | 0.031 | 1 | 0.860 |

**Table 8: Model summary results from model for morph-specific survival**

| **Explanatory variable** | **est.** | **s.e.** | **z-value** | **p-value** |
| --- | --- | --- | --- | --- |
| Intercept | 1.962 | 0.172 | 11.409 | **<0.001** |
| temperatureheatwave | -0.659 | 0.180 | -3.656 | **<0.001** |
| pheromonetreatment | 0.689 | 0.157 | 4.401 | **<0.001** |
| morphscrambler | -0.193 | 0.093 | -2.085 | **0.037** |

**Table 9: Results from fitting a GLMM and LMM to predict female proportion and female number respectively in the second counts.**
*The factors and interactions retained in the final model are denoted in bold. χ², F values, d.f., and p-values are from likelihood ratio tests comparing models with and without the variable in question.*

| **Source of variation** | **Female Proportion** | | | **Number of Females** | | |
| --- | --- | --- | --- | --- | --- | --- |
|  | χ² | d.f. | p | F value | d.f. | **p-value** |
| **temperature** | **14.153** | **1** | **<0.001** | **48.049** | **1** | **<0.001** |
| **pheromone** | **33.856** | **1** | **<0.001** | - | - | - |
| **generation** | **21.913** | **1** | **<0.001** | - | - | - |
| temperature * generation | 2.144 | 1 | 0.143 | 2.501 | 1 | 0.115 |
| temperature * pheromone | 1.373 | 1 | 0.241 | 3.133 | 1 | 0.085 |
| **pheromone * generation** | 1.180 | 1 | 0.274 | **7.456** | **1** | **0.007** |
| temperature*pheromone*generation | 0.125 | 1 | 0.724 | 0.033 | 1 | 0.857 |

**Table 10: Model summary results from model for proportion of females and number of females in the second counts**

| **Explanatory variable** | | **Proportion of Females** | | | | **Number of Females** | | | |
| --- | --- | --- | --- | --- | --- | --- | --- | --- | --- |
|  | est. | | s.e. | z-value | p-value | est. | s.e. | t-value | p-value |
| Intercept | 0.653 | | 0.058 | 11.092 | <0.001 | 23.616 | 0.979 | 24.119 | **<0.001** |
| temperatureheatwave | -0.211 | | 0.052 | -4.094 | <0.001 | -5.757 | 0.831 | -6.932 | **<0.001** |
| pheromonetreatment | -0.339 | | 0.048 | -7.006 | <0.001 | 1.9033 | 1.105 | 1.722 | **0.087** |
| generation | -0.040 | | 0.009 | -4.674 | <0.001 | -0.510 | 0.164 | -3.117 | **0.002** |
| pheromonetreatment:generation |  | |  |  |  | -0.644 | 0.236 | -2.731 | **0.007** |

**R script**

rm(list = ls(all = TRUE))

ls()

# packages ----------------------------------------------------------------

library(readxl)

library(glmmTMB)

library(ggplot2)

library(bbmle)

library(pscl)

library(lmerTest)

library(DHARMa)

library(ggpubr)

library(ggh4x)

library(patchwork)

library(tidyr)

library(tidyverse)

library(dplyr)

setwd("~/Desktop/extinction_sancassania/final")

# function for overdispersion ---------------------------------------------

overdisp_fun <- function(model) {

rdf <- df.residual(model)

rp <- residuals(model, type = "pearson")

Pearson.chisq <- sum(rp ^ 2)

prat <- Pearson.chisq / rdf

pval <- pchisq(Pearson.chisq, df = rdf, lower.tail = FALSE)

c(

chisq = Pearson.chisq,

ratio = prat,

rdf = rdf,

p = pval

)

}

# Fighter proportions- adult survival -------------------------------------

rm(list = ls(all = TRUE))

ls()

san_morph = read_excel("SS_heat_demography.xlsx", sheet = "morph")

san_morph$line = as.factor(san_morph$line)

san_morph$Pheromone = as.factor(san_morph$Pheromone)

san_morph$Temperature <- as.factor(san_morph$Temperature)

san_morph$fig = as.numeric(san_morph$fig)

san_morph$scr = as.numeric(san_morph$scr)

san_morph$female = as.numeric(san_morph$female)

san_morph$gen = as.numeric(san_morph$gen)

san_morph_notrit = subset(san_morph, gen != 8)

san_morph_notrit = subset(san_morph_notrit, trito == 0)

san_morph_notrit$Temperature <- factor(san_morph_notrit$Temperature, levels = c("Stable", "Heatwave"))

morph_wo_trit <- glmer(

cbind(fig, scr) ~ (Temperature + Pheromone + count +

as.numeric(gen)) ^ 3 + (1 |line),

data = san_morph_notrit,

family = binomial,

control = glmerControl(optimizer = 'bobyqa', optCtrl = list(maxfun = 100000))

)

drop1(morph_wo_trit, test = "Chi")

morph_wo_trit <- update(morph_wo_trit,~.-Pheromone:count:as.numeric(gen))

drop1(morph_wo_trit, test = "Chi")

morph_wo_trit <- update(morph_wo_trit,~.-Temperature:count:as.numeric(gen))

drop1(morph_wo_trit, test = "Chi")

morph_wo_trit <- update(morph_wo_trit,~.-Temperature:Pheromone:count)

drop1(morph_wo_trit, test = "Chi")

morph_wo_trit <- glmer(

cbind(fig, scr) ~ (Temperature + Pheromone + count +

as.numeric(gen)) ^ 2 + (1 |line),

data = san_morph_notrit,

family = binomial,

control = glmerControl(optimizer = 'bobyqa', optCtrl = list(maxfun = 100000))

)

drop1(morph_wo_trit, test = "Chi")

morph_wo_trit <- update(morph_wo_trit,~.-Temperature:Pheromone)

drop1(morph_wo_trit, test = "Chi")

morph_wo_trit <- update(morph_wo_trit,~.-Pheromone:as.numeric(gen))

drop1(morph_wo_trit, test = "Chi")

morph_wo_trit <- update(morph_wo_trit,~.-Temperature:count)

drop1(morph_wo_trit, test = "Chi")

morph_wo_trit <- update(morph_wo_trit,~.-count:as.numeric(gen))

drop1(morph_wo_trit, test = "Chi")

morph_wo_trit <- update(morph_wo_trit,~.-Pheromone:count)

drop1(morph_wo_trit, test = "Chi")

morph_wo_trit <- update(morph_wo_trit,~.-count)

drop1(morph_wo_trit, test = "Chi")

#check for overdispersion

overdisp_fun(morph_wo_trit)#ratio=1.44

#diagnostic plots

simulateResiduals(fittedModel = morph_wo_trit, plot = TRUE)

#accounting for overdispersion using betabinomial error distribution

morph_beta = glmmTMB(cbind(fig, scr) ~ (Temperature + Pheromone + count + as.numeric (gen))^3 + (1|line), data = san_morph_notrit, family = betabinomial,

control=glmmTMBControl(optCtrl=list(iter.max=1000000)))

drop1(morph_beta,test="Chi")

morph_beta <- update(morph_beta,~.-Pheromone:count:as.numeric(gen))

drop1(morph_beta, test = "Chi")

morph_beta <- update(morph_beta,~.-Temperature:count:as.numeric(gen))

drop1(morph_beta, test = "Chi")

morph_beta <- update(morph_beta,~.-Temperature:Pheromone:count)

drop1(morph_beta, test = "Chi")

morph_beta = glmmTMB(cbind(fig, scr) ~ (Temperature + Pheromone + count + as.numeric (gen))^2 + (1|line), data = san_morph_notrit, family = betabinomial,

control=glmmTMBControl(optCtrl=list(iter.max=1000000)))

drop1(morph_beta,test="Chi")

morph_beta <- update(morph_beta,~.-Temperature:count)

drop1(morph_beta, test = "Chi")

morph_beta <- update(morph_beta,~.-Pheromone:as.numeric(gen))

drop1(morph_beta, test = "Chi")

morph_beta <- update(morph_beta,~.-count:as.numeric(gen))

drop1(morph_beta, test = "Chi")

morph_beta <- update(morph_beta,~.-Temperature:Pheromone)

drop1(morph_beta, test = "Chi")

morph_beta <- update(morph_beta,~.-Pheromone:count)

drop1(morph_beta, test = "Chi")

morph_beta = glmmTMB(cbind(fig, scr) ~ (Temperature + Pheromone + as.numeric(gen))^2

-Pheromone:as.numeric(gen)

-Temperature:Pheromone + (1|line), data = san_morph_notrit, family = betabinomial,

control=glmmTMBControl(optCtrl=list(iter.max=1000000)))

drop1(morph_beta,test="Chi")

#diagnostic plots

simulateResiduals(fittedModel = morph_beta, plot = TRUE)

summary(morph_beta)

#graph for fighter proportion

average_counts <- san_morph_notrit %>%

filter(count %in% c("First adult count", "Second adult count")) %>%

group_by(line, gen) %>%

summarize(

average_fig = mean(fig, na.rm = TRUE),

average_scr = mean(scr, na.rm = TRUE),

Pheromone = first(Pheromone),

Temperature = first(Temperature),

.groups = 'drop'

)

average_counts$gen<- as.numeric(average_counts$gen)

# Update Pheromone labels to match your preferences

average_counts$fig_prop <- (average_counts$average_fig / (average_counts$average_fig + average_counts$average_scr))

# write_xlsx(average_counts, path = "average fighter proportion.xlsx")

morph_dummy <- data.frame(

gen = rep((0:7), times = 4),

Temperature = rep(c("Stable", "Heatwave"), each = 16),

#count = rep(c("First adult count", "Second adult count"), each = 16, times = ),

Pheromone = rep(c("Control", "Treatment"), each = 8, times = 2)

)

predicted_morph <- predict(

morph_beta,

newdata = data.frame(morph_dummy, line = NA),

se.fit = TRUE,

type = "response"

)

# Create predicted values dataframe with the CI bounds

predicted_values_morph <- data.frame(morph_dummy,

predict = predicted_morph$fit,

se = predicted_morph$se.fit)

predicted_values_morph$lowerCI <- predicted_values_morph$predict - 1.96 * predicted_values_morph$se

predicted_values_morph$upperCI <- predicted_values_morph$predict + 1.96 * predicted_values_morph$se

predicted_values_morph$Temperature <- factor(predicted_values_morph$Temperature, levels = c("Stable", "Heatwave"))

fig_2 <- ggplot(average_counts,

aes(

x = as.numeric(gen),

y = fig_prop,

shape = Pheromone, # Different shapes for points

linetype = Pheromone, # Different line types for trends

colour = Pheromone,

fill = Pheromone

)) +

geom_point(size = 2.2, alpha = 0.3, position = position_dodge(width =0.40)) +

scale_x_continuous(breaks = c(0, 1, 2, 3, 4, 5, 6, 7)) +

scale_x_continuous(breaks = c(0, 1, 2, 3, 4, 5, 6, 7)) +

scale_color_manual(values = c("#1b9e77", "#d95f02"),

name = "Pheromone",

labels = c("Pheromone control", "Pheromone treatment")) +

scale_fill_manual(values = c("#1b9e77", "#d95f02"),

name = "Pheromone",

labels = c("Pheromone control", "Pheromone treatment")) +

scale_shape_manual(values = c(17, 16),

name = "Pheromone",

labels = c("Pheromone control", "Pheromone treatment")) +

scale_linetype_manual(values = c("solid", "dashed"),

name = "Pheromone",

labels = c("Pheromone control", "Pheromone treatment")) +

# Add ribbon for CI shading

geom_ribbon(data = predicted_values_morph,

aes(x = gen, ymin = lowerCI, ymax = upperCI, fill = Pheromone),

alpha = 0.3, inherit.aes = FALSE) +

# Add the line for predicted values

geom_line(data = predicted_values_morph,

aes(x = gen, y = predict, colour = Pheromone, linetype = Pheromone), linewidth = 1) +

facet_grid(~Temperature) +

labs(x = "Generation", y = "Proportion of fighters") +

theme_light() +

theme(panel.grid.minor = element_blank(),

legend.key.size = unit(1, 'cm'),

legend.key.height = unit(1, 'cm'),

legend.key.width = unit(1, 'cm'),

legend.title = element_text(size=14, face = "bold"),

legend.text = element_text(size=14),

legend.position = "bottom",

axis.text = element_text(size = 14),

axis.title = element_text(size = 14, face = "bold"),

strip.text.x = element_text(size = 14, color = "black"),

strip.text.y = element_text(size = 14, color = "black"),

strip.background = element_rect(

color = "black",

fill = "#F2F4B5",

size = 1.5,

linetype = "solid"

)

)

# Display the plot

fig_2

ggsave(

filename = "fig_2.pdf",

plot = fig_2,

dpi = 900

)

# Population level individual survival ------------------------------------

san_ext <- read_excel("SS_heat_demography.xlsx", sheet = "survival")

san_ext = subset(san_ext, generation != 8)

san_ext$line = as.factor(san_ext$line)

san_ext$morph = as.factor(san_ext$Pheromone)

san_ext$juvenile = as.numeric(san_ext$juvenile)

san_ext$ad_total_I = as.numeric(san_ext$ad_total_I)

san_ext$ad_total_II = as.numeric(san_ext$ad_total_II)

san_ext$Temperature <- factor(san_ext$Temperature, levels = c("Stable", "Heatwave"))

# Survival from juvenile to first adult count -----------------------------

san_juv_sur <- san_ext %>%

mutate(across(c(ad_total_I, juvenile), ~ ifelse(generation == 0, NA, .)))

#san_juv_sur<- subset(san_juv, generation != 0)

juv_sur = glmer(

cbind(ad_total_I, juvenile - ad_total_I) ~ (Temperature + Pheromone +

as.numeric(generation)) ^ 3 + (1 | line),

data = san_juv_sur,

family = binomial,

control = glmerControl(optimizer = 'bobyqa', optCtrl = list(maxfun = 100000))

)

drop1(juv_sur, test = "Chi")

juv_sur = glmer(

cbind(ad_total_I, juvenile - ad_total_I) ~ (Temperature + Pheromone +

as.numeric(generation)) ^ 2 + (1 | line),

data = san_juv_sur,

family = binomial,

control = glmerControl(optimizer = 'bobyqa', optCtrl = list(maxfun = 100000))

)

drop1(juv_sur, test = "Chi")

juv_sur = glmer(cbind(ad_total_I, juvenile - ad_total_I) ~ (Temperature + Pheromone +

as.numeric(generation)) ^ 2 - Temperature:Pheromone + (1 | line),

data = san_juv_sur,

family = binomial,

control = glmerControl(optimizer = 'bobyqa', optCtrl = list(maxfun = 100000))

)

drop1(juv_sur, test = "Chi")

#diagnostic plots

simulateResiduals(fittedModel = juv_sur, plot = TRUE)

#checking for overdispersion

overdisp_fun(juv_sur) # ratio= 3.58

#accounting for over dispersion using beta-binomial error distribution

juv_sur_beta = glmmTMB(

cbind(ad_total_I, juvenile - ad_total_I) ~ (Temperature + Pheromone +

as.numeric(generation))^3 + (1 | line),

data = san_juv_sur,

family = betabinomial,

control = glmmTMBControl(optCtrl = list(iter.max =

1000000)))

drop1(juv_sur_beta, test = "Chi")

juv_sur_beta = glmmTMB(

cbind(ad_total_I, juvenile - ad_total_I) ~ (Temperature + Pheromone +

as.numeric(generation))^2 + (1 | line),

data = san_juv_sur,

family = betabinomial,

control = glmmTMBControl(optCtrl = list(iter.max =

1000000)))

drop1(juv_sur_beta, test = "Chi")

juv_sur_beta = glmmTMB(

cbind(ad_total_I, juvenile - ad_total_I) ~ (Temperature + Pheromone +

as.numeric(generation)) ^2 -Temperature:as.numeric(generation)+ (1 | line),

data = san_juv_sur,

family = betabinomial,

control = glmmTMBControl(optCtrl = list(iter.max =

1000000))

)

drop1(juv_sur_beta, test = "Chi")

juv_sur_beta = glmmTMB(

cbind(ad_total_I, juvenile - ad_total_I) ~ (Temperature + Pheromone +

as.numeric(generation)) ^2 -Temperature:as.numeric(generation) - Temperature:Pheromone + (1 | line),

data = san_juv_sur,

family = betabinomial,

control = glmmTMBControl(optCtrl = list(iter.max =

1000000)) # for graph

)

drop1(juv_sur_beta, test = "Chi")

juv_sur_beta = glmmTMB(

cbind(ad_total_I, juvenile - ad_total_I) ~ (Pheromone +

as.numeric(generation)) ^2 + (1 | line),

data = san_juv_sur,

family = betabinomial,

control = glmmTMBControl(optCtrl = list(iter.max =

1000000))

)

drop1(juv_sur_beta, test = "Chi")

#diagnostic plots

simulateResiduals(fittedModel = juv_sur_beta, plot = TRUE)

summary(juv_sur_beta) #best

#plotting the predictions from juvenile survival/ pre heat stress survival from juv_sur_beta

#create a data frame to store the predictions

juv_dummy <- data.frame(

generation = rep((0:7), times = 4),

Temperature = rep(c("Stable", "Heatwave"), each = 16),

Pheromone = rep(c("Control", "Treatment"), each = 8, times = 2)

)

# Predict juvenile survival

predicted_juv <- predict(

juv_sur_beta,

newdata = data.frame(juv_dummy, line = NA),

se.fit = TRUE,

type = "response"

)

# Store predicted values and CIs

predicted_values_juv <- data.frame(juv_dummy, predict = predicted_juv$fit, se = predicted_juv$se.fit)

predicted_values_juv$lowerCI <- predicted_values_juv$predict - 1.96 * predicted_values_juv$se

predicted_values_juv$upperCI <- predicted_values_juv$predict + 1.96 * predicted_values_juv$se

predicted_values_juv$Temperature <- factor(predicted_values_juv$Temperature, levels = c("Stable", "Heatwave"))

# Calculate the proportion of juveniles survived (raw data)

san_juv_sur$juv_prop = san_juv_sur$ad_total_I / san_juv_sur$juvenile

fig_3a <- ggplot(san_juv_sur,

aes(

x = as.numeric(generation),

y = juv_prop,

shape = Pheromone, # Set shape for points

linetype = Pheromone, # Set line type for lines

colour = Pheromone,

fill = Pheromone

)) +

geom_point(size = 2.2, alpha = 0.3, position = position_dodge(width =0.40)) +

scale_x_continuous(breaks = c(0, 1, 2, 3, 4, 5, 6, 7)) +

scale_color_manual(values = c("#1b9e77", "#d95f02"),

name = "Pheromone",

labels = c("Pheromone control", "Pheromone treatment")) +

scale_fill_manual(values = c("#1b9e77", "#d95f02"),

name = "Pheromone",

labels = c("Pheromone control", "Pheromone treatment")) +

scale_shape_manual(values = c(17, 16),

name = "Pheromone",

labels = c("Pheromone control", "Pheromone treatment")) +

scale_linetype_manual(values = c("solid", "dashed"),

name = "Pheromone",

labels = c("Pheromone control", "Pheromone treatment")) +

# Add ribbon for CI shading (excluding generation == 0)

geom_ribbon(data = predicted_values_juv %>% filter(generation > 0),

aes(x = generation, ymin = lowerCI, ymax = upperCI, fill = Pheromone),

alpha = 0.4, inherit.aes = FALSE) +

# Add the line for predicted values (excluding generation == 0)

geom_line(data = predicted_values_juv %>% filter(generation > 0),

aes(x = generation, y = predict, colour = Pheromone, linetype = Pheromone), linewidth = 1) +

facet_wrap(~ Temperature) +

labs(x = "Generation", y = "Early survival") +

theme_light() +

theme(panel.grid.minor = element_blank()) +

theme(

axis.text = element_text(size = 16),

axis.title = element_text(size = 16, face = "bold"),

#axis.title.x = element_blank(), axis.text.x = element_blank(),

strip.text.x = element_text(size = 16, color = "black"),

strip.text.y = element_text(size = 16, color = "black"),

strip.background = element_rect(

color = "black", fill = "#F2F4B5", size = 1.5, linetype = "solid"

),

legend.text = element_text(size = 15),

legend.title = element_text(size = 18, face = "bold"),

legend.key.height = unit(1.0, "cm"),

legend.key.width = unit(1.0, "cm")

)

# Display the plot

fig_3a

# First adult count to second adult count survival ------------------------

adult_sur = glmer(

cbind(ad_total_II, ad_total_I - ad_total_II) ~ (Temperature + Pheromone +

as.numeric(generation)) ^ 3 + (1 | line),

data = san_ext,

family = binomial,

control = glmerControl(optimizer = 'bobyqa', optCtrl = list(maxfun = 100000))

)

drop1(adult_sur, test = "Chi")

adult_sur = glmer(

cbind(ad_total_II, ad_total_I - ad_total_II) ~ (Temperature + Pheromone +

as.numeric(generation)) ^ 2 + (1 | line),

data = san_ext,

family = binomial,

control = glmerControl(optimizer = 'bobyqa', optCtrl = list(maxfun = 100000))

)

drop1(adult_sur, test = "Chi")

adult_sur <- update(adult_sur,~.-Temperature:as.numeric(generation))

drop1(adult_sur, test = "Chi")

adult_sur <- update(adult_sur,~.-Temperature:Pheromone)

drop1(adult_sur, test = "Chi")

adult_sur <- update(adult_sur,~.-Pheromone:as.numeric(generation))

drop1(adult_sur, test = "Chi")

#check for overdispersion

overdisp_fun(adult_sur) # ratio= 2.4

#diagnostic plots

simulateResiduals(fittedModel = adult_sur, plot = TRUE)

#accounting for over dispersion using beta binomial error distribution

adult_sur_beta = glmmTMB(

cbind(ad_total_II, ad_total_I - ad_total_II) ~ (Temperature + Pheromone +

as.numeric(generation)) ^ 3 + (1 |line),

data = san_ext,

family = betabinomial,

control = glmmTMBControl(optCtrl = list(iter.max =

1000000))

)

drop1(adult_sur_beta, test = "Chi")

adult_sur_beta = glmmTMB(

cbind(ad_total_II, ad_total_I - ad_total_II) ~ (Temperature + Pheromone +

as.numeric(generation)) ^ 2 + (1 |line),

data = san_ext,

family = betabinomial,

control = glmmTMBControl(optCtrl = list(iter.max =

1000000))

)

drop1(adult_sur_beta, test = "Chi")

adult_sur_beta <- update(adult_sur_beta,~.-Temperature:as.numeric(generation))

drop1(adult_sur_beta, test = "Chi")

adult_sur_beta <- update(adult_sur_beta,~.-Temperature:Pheromone)

drop1(adult_sur_beta, test = "Chi")

adult_sur_beta = glmmTMB(

cbind(ad_total_II, ad_total_I - ad_total_II) ~ (Temperature + Pheromone +

as.numeric(generation)) ^ 2

- Temperature:as.numeric(generation) -Temperature:Pheromone

-Pheromone:as.numeric(generation)+ (1 |line),

data = san_ext,

family = betabinomial,

control = glmmTMBControl(optCtrl = list(iter.max =

1000000))

)

drop1(adult_sur_beta, test = "Chi")

#diagnostic plots

simulateResiduals(fittedModel = adult_sur_beta, plot = TRUE)

summary(adult_sur_beta)

#adults survived proportion

san_ext$adult_prop = san_ext$ad_total_II / san_ext$ad_total_I

# Prepare dummy data for predictions

adult_dummy <- data.frame(

generation = rep((0:7), times = 4),

Temperature = rep(c("Stable", "Heatwave"), each = 16),

Pheromone = rep(c("Control", "Treatment"), each = 8, times = 2)

)

# Make predictions from the model (adult_sur_beta)

predicted_adult <- predict(

adult_sur_beta,

data.frame(adult_dummy, line = NA),

se.fit = TRUE,

type = "response"

)

# Store predicted values and their standard errors

predicted_values_adult <- data.frame(adult_dummy, predict = predicted_adult$fit, se = predicted_adult$se.fit)

predicted_values_adult$lowerCI <- predicted_values_adult$predict - 1.96 * predicted_values_adult$se

predicted_values_adult$upperCI <- predicted_values_adult$predict + 1.96 * predicted_values_adult$se

san_ext$Temperature <- factor(san_ext$Temperature, levels = c("Stable", "Heatwave"))

predicted_values_adult$Temperature <- factor(predicted_values_adult$Temperature, levels = c("Stable", "Heatwave"))

# Plot adult survival with predicted values and confidence intervals

fig_3b <- ggplot(san_ext,

aes(

x = as.numeric(generation),

y = adult_prop,

shape = Pheromone, # Set shape for points

linetype = Pheromone, # Set line type for lines

colour = Pheromone,

fill = Pheromone

)) +

geom_point(size = 2.2, alpha = 0.3, position = position_dodge(width =0.40)) +

scale_x_continuous(breaks = c(0, 1, 2, 3, 4, 5, 6, 7)) +

scale_color_manual(values = c("#1b9e77", "#d95f02"),

name = "Pheromone",

labels = c("Pheromone control", "Pheromone treatment")) +

scale_fill_manual(values = c("#1b9e77", "#d95f02"),

name = "Pheromone",

labels = c("Pheromone control", "Pheromone treatment")) +

scale_shape_manual(values = c(17, 16),

name = "Pheromone",

labels = c("Pheromone control", "Pheromone treatment")) +

scale_linetype_manual(values = c("solid", "dashed"),

name = "Pheromone",

labels = c("Pheromone control", "Pheromone treatment")) +

# Add ribbon for CI shading

geom_ribbon(data = predicted_values_adult,

aes(x = generation, ymin = lowerCI, ymax = upperCI, fill = Pheromone),

alpha = 0.4, inherit.aes = FALSE) +

# Add the line for predicted values

geom_line(data = predicted_values_adult,

aes(x = generation, y = predict, colour = Pheromone, linetype = Pheromone), linewidth = 1) +

facet_wrap(~ Temperature) +

labs(x = "Generation", y = "Late survival") +

theme_light() +

theme(

panel.grid.minor = element_blank(),

axis.text = element_text(size = 16),

axis.title = element_text(size = 16, face = "bold"),

#axis.title.x = element_blank(),

#axis.text.x = element_blank(),

strip.text.x = element_text(size = 16, color = "black"),

strip.text.y = element_text(size = 16, color = "black"),

strip.background = element_rect(

color = "black", fill = "#F2F4B5", size = 1.5, linetype = "solid"

),

legend.text = element_text(size = 15),

legend.title = element_text(size = 18, face = "bold"),

legend.key.height = unit(1.0, "cm"),

legend.key.width = unit(1.0, "cm")

)

# Display the plot

fig_3b

fig_3<-ggarrange(

fig_3a,

fig_3b,

nrow = 2,

labels = c('a', 'b'),

common.legend = TRUE,

legend = "bottom",

font.label = list(size = 20, face = "bold")

)

fig_3

ggsave(

filename = "fig_3.pdf",

plot = fig_3,

dpi = 800,

)

# Sex specific adult survival ---------------------------------------------

rm(list = ls(all = TRUE))

ls()

san_sex = read_excel("SS_heat_demography.xlsx", sheet = "sex")

san_sex$line = as.factor(san_sex$line)

san_sex$Pheromone = as.factor(san_sex$Pheromone)

san_sex$Temperature = as.factor(san_sex$Temperature)

san_sex$sex = as.factor(san_sex$sex)

san_sex$ad_total_I = as.numeric(san_sex$ad_total_I)

san_sex$ad_total_II = as.numeric(san_sex$ad_total_II)

san_sex_notrit = subset(san_sex, trito == 0)

san_sex_notrit = subset(san_sex, generation != 8)

san_sex_notrit$Temperature <- factor(san_sex_notrit$Temperature, levels = c("Stable", "Heatwave"))

sex_sur = glmer(

cbind(ad_total_II, ad_total_I - ad_total_II) ~ (Temperature + Pheromone + sex +

as.numeric(generation))^3 + Temperature:sex:as.numeric(generation) + (1 |line),

data = san_sex_notrit,

family = binomial,

control = glmerControl(optimizer = 'bobyqa', optCtrl = list(maxfun = 100000))

)

drop1(sex_sur, test = "Chi")

sex_sur <- update(sex_sur,~.-Temperature:Pheromone:as.numeric(generation))

drop1(sex_sur, test = "Chi")

sex_sur <- update(sex_sur,~.-Temperature:Pheromone:sex)

drop1(sex_sur, test = "Chi")

sex_sur <- update(sex_sur,~.-Temperature:Pheromone)

drop1(sex_sur, test = "Chi")

overdisp_fun(sex_sur)#ratio=1.87

#diagnostic plots

simulateResiduals(fittedModel = sex_sur, plot = TRUE)

#dealing with overdispersion

sex_sur_beta = glmmTMB(

cbind(ad_total_II, ad_total_I - ad_total_II) ~ (Temperature + Pheromone + sex +

as.numeric(generation)) ^3+ Temperature:sex:as.numeric(generation) + (1 | line),

data = san_sex_notrit,

family = betabinomial,

control = glmmTMBControl(optCtrl = list(iter.max =

10000)))

drop1(sex_sur_beta, test = "Chi")

sex_sur_beta <- update(sex_sur_beta,~.-Temperature:Pheromone:sex)

drop1(sex_sur_beta, test = "Chi")

sex_sur_beta <- update(sex_sur_beta,~.-Temperature:Pheromone:as.numeric(generation))

drop1(sex_sur_beta, test = "Chi")

sex_sur_beta <- update(sex_sur_beta,~.-Pheromone:sex:as.numeric(generation))

drop1(sex_sur_beta, test = "Chi")

sex_sur_beta <- update(sex_sur_beta,~.-Temperature:sex:as.numeric(generation))

drop1(sex_sur_beta, test = "Chi")

sex_sur_beta <- update(sex_sur_beta,~.-Temperature:Pheromone)

drop1(sex_sur_beta, test = "Chi")

sex_sur_beta = glmmTMB(

cbind(ad_total_II, ad_total_I - ad_total_II) ~ (Temperature + Pheromone + sex +

as.numeric(generation)) ^2

-Temperature:Pheromone -Pheromone:as.numeric(generation) + (1 |line),

data = san_sex_notrit,

family = betabinomial,

control = glmmTMBControl(optCtrl = list(iter.max =

10000))

)

drop1(sex_sur_beta, test = "Chi")

#diagnostic plots

simulateResiduals(fittedModel = sex_sur_beta, plot = TRUE)

san_sex_notrit$sex_prop_sur = san_sex_notrit$ad_total_II / san_sex_notrit$ad_total_I

summary(sex_sur_beta)

sex_dummy <- data.frame(

generation = rep((0:7), times = 8),

Temperature = rep(c("Stable", "Heatwave"), each = 32),

sex = rep(c("male", "female"), each = 16, times = 2),

Pheromone = rep(c("Control", "Treatment"), each = 8, times = 4)

)

predicted_sex <- predict(

sex_sur_beta,

newdata = data.frame(sex_dummy, line = NA),

se.fit = TRUE,

type = "response"

)

# Create predicted values dataframe with the CI bounds

predicted_values_sex <- data.frame(sex_dummy, predict = predicted_sex$fit, se = predicted_sex$se.fit)

predicted_values_sex$lowerCI <- predicted_values_sex$predict - 1.96 * predicted_values_sex$se

predicted_values_sex$upperCI <- predicted_values_sex$predict + 1.96 * predicted_values_sex$se

predicted_values_sex$Temperature <- factor(predicted_values_sex$Temperature, levels = c("Stable", "Heatwave"))

# Plot sex specific survival with predicted values and confidence intervals

fig_4 <- ggplot(san_sex_notrit,

aes(

x = as.numeric(generation),

y = sex_prop_sur,

shape = Pheromone, # Different shapes for points

linetype = Pheromone, # Different line types for trends

colour = Pheromone,

fill = Pheromone

)) +

geom_point(size = 2.2, alpha = 0.3, position = position_dodge(width =0.40)) +

scale_x_continuous(breaks = c(0, 1, 2, 3, 4, 5, 6, 7)) +

scale_color_manual(values = c("#1b9e77", "#d95f02"),

name = "Pheromone",

labels = c("Pheromone control", "Pheromone treatment")) +

scale_fill_manual(values = c("#1b9e77", "#d95f02"),

name = "Pheromone",

labels = c("Pheromone control", "Pheromone treatment")) +

scale_shape_manual(values = c(17, 16),

name = "Pheromone",

labels = c("Pheromone control", "Pheromone treatment")) +

scale_linetype_manual(values = c("solid", "dashed"),

name = "Pheromone",

labels = c("Pheromone control", "Pheromone treatment")) +

# Add ribbon for CI shading

geom_ribbon(data = predicted_values_sex,

aes(x = generation, ymin = lowerCI, ymax = upperCI, fill = Pheromone),

alpha = 0.4, inherit.aes = FALSE) +

# Add the line for predicted values

geom_line(data = predicted_values_sex,

aes(x = generation, y = predict, colour = Pheromone, linetype = Pheromone), linewidth = 1) +

facet_grid(Temperature ~ sex) +

labs(x = "Generation", y = " Late survival") +

theme_light() +

theme(panel.grid.minor = element_blank(),

legend.key.size = unit(1, 'cm'),

legend.key.height = unit(1, 'cm'),

legend.key.width = unit(1, 'cm'),

legend.title = element_text(size=14, face = "bold"),

legend.text = element_text(size=14),

legend.position = "bottom",

axis.text = element_text(size = 14),

axis.title = element_text(size = 14, face = "bold"),

strip.text.x = element_text(size = 14, color = "black"),

strip.text.y = element_text(size = 14, color = "black"),

strip.background = element_rect(

color = "black",

fill = "#F2F4B5",

size = 1.5,

linetype = "solid"

)

)

# Display the graph

fig_4

ggsave(

filename = "fig_4.pdf",

plot = fig_4,

dpi = 900

)

# morph specific survival -------------------------------------

rm(list = ls(all = TRUE))

ls()

san_morph = read_excel("SS_heat_demography.xlsx", sheet = "morph")

san_morph$line = as.factor(san_morph$line)

san_morph$Pheromone = as.factor(san_morph$Pheromone)

san_morph$Temperature <- as.factor(san_morph$Temperature)

san_morph$fig = as.numeric(san_morph$fig)

san_morph$scr = as.numeric(san_morph$scr)

san_morph$gen = as.numeric(san_morph$gen)

san_morph_notrit = subset(san_morph, gen != 8)

san_morph_notrit = subset(san_morph_notrit, trito == 0)

san_morph_notrit$Temperature <- factor(san_morph_notrit$Temperature, levels = c("Stable", "Heatwave"))

morph_sur_mod = glmer(

cbind(`Second adult count`, (`First adult count` - `Second adult count`)) ~ (Temperature + Pheromone + morph_type +

as.numeric(gen))^2 + (1 |line),

data = morph_sur,

family = binomial,

control = glmerControl(optimizer = 'bobyqa', optCtrl = list(maxfun = 100000))

)

drop1(morph_sur_mod, test = "Chi")

morph_sur_mod <- update(morph_sur_mod,~.-Temperature:morph_type)

drop1(morph_sur_mod, test = "Chi")

morph_sur_mod <- update(morph_sur_mod,~.-Temperature:as.numeric(gen))

drop1(morph_sur_mod, test = "Chi")

morph_sur_mod <- update(morph_sur_mod,~.-Pheromone:morph_type)

drop1(morph_sur_mod, test = "Chi")

morph_sur_mod <- update(morph_sur_mod,~.-morph_type:as.numeric(gen))

drop1(morph_sur_mod, test = "Chi")

morph_sur_mod <- update(morph_sur_mod,~.-Pheromone:as.numeric(gen))

drop1(morph_sur_mod, test = "Chi")

morph_sur_mod <- update(morph_sur_mod,~.-Temperature:Pheromone)

drop1(morph_sur_mod, test = "Chi")

morph_sur_mod = glmer(

cbind(`Second adult count`, (`First adult count` - `Second adult count`)) ~ Temperature + Pheromone + morph_type + (1 |line),

data = morph_sur,

family = binomial,

control = glmerControl(optimizer = 'bobyqa', optCtrl = list(maxfun = 100000))

)

drop1(morph_sur_mod, test = "Chi")

overdisp_fun(morph_sur_mod) #1.38

#diagnostic plots

simulateResiduals(fittedModel = morph_sur_mod, plot = TRUE)

testDispersion(simulateResiduals(fittedModel = morph_sur_mod))

summary(morph_sur_mod)

morph_sur$morph_prop_sur = morph_sur$`Second adult count` / morph_sur$`First adult count`

# Plot sex specific survival with predicted values and confidence intervals

fig_5 <- ggplot(morph_sur,

aes(

x = morph_type,

y = morph_prop_sur,

shape = Pheromone,

colour = Pheromone,

fill = Pheromone

)) +

# Points with jitter and dodge

geom_point(size = 3, alpha = 0.5,

position = position_jitterdodge(

jitter.width = 0.4, # Adjust jitter width for spread

dodge.width = 0.75 # Match dodge width with boxplot

)) +

# Boxplot with thickened median

geom_boxplot(data = morph_sur,

aes(x = morph_type, y = morph_prop_sur, fill = Pheromone),

alpha = 0.4,

fatten = 3, outlier.shape = NA) + # Thicken median line

stat_summary(aes(group = Pheromone),

fun = mean,

geom = "point",

color = "black",

size = 3,

shape = 18,

position = position_dodge(width = 0.75)) + # Match dodge width

# Color scale

scale_color_manual(values = c("#1b9e77", "#d95f02"),

name = "Pheromone",

labels = c("Pheromone control", "Pheromone treatment")) +

# Fill scale

scale_fill_manual(values = c("#1b9e77", "#d95f02"),

name = "Pheromone",

labels = c("Pheromone control", "Pheromone treatment")) +

# Shape scale

scale_shape_manual(values = c(17, 16),

name = "Pheromone",

labels = c("Pheromone control", "Pheromone treatment")) +

# Rename x-axis categories

scale_x_discrete(labels = c("fig" = "Fighters", "scr" = "Scramblers")) +

# Facet by Temperature

facet_grid(~Temperature) +

labs(x = "Morph", y = "Late survival") +

theme_light() +

theme(

panel.grid.minor = element_blank(),

legend.key.size = unit(1, 'cm'),

legend.key.height = unit(1, 'cm'),

legend.key.width = unit(1, 'cm'),

legend.title = element_text(size = 14, face = "bold"),

legend.text = element_text(size = 14),

legend.position = "bottom",

axis.text = element_text(size = 14),

axis.title = element_text(size = 14, face = "bold"),

strip.text.x = element_text(size = 14, color = "black"),

strip.text.y = element_text(size = 14, color = "black"),

strip.background = element_rect(

color = "black",

fill = "#F2F4B5",

size = 1.5,

linetype = "solid"

)

)

# Display the graph

print(fig_5)

ggsave(

filename = "fig_5.pdf",

plot = fig_5,

dpi = 900

)

# Female proportion with trito only second count --------------------------

rm(list = ls(all = TRUE))

ls()

san_morph = read_excel("SS_heat_demography.xlsx", sheet = "morph")

san_morph$line = as.factor(san_morph$line)

san_morph$Pheromone = as.factor(san_morph$Pheromone)

san_morph$Temperature <- as.factor(san_morph$Temperature)

san_morph$fig = as.numeric(san_morph$fig)

san_morph$scr = as.numeric(san_morph$scr)

san_morph$female = as.numeric(san_morph$female)

san_morph$gen = as.numeric(san_morph$gen)

san_fem_prop = subset(san_morph, gen != 8 & count== "Second adult count")

san_fem_prop$male<- san_fem_prop$fig + san_fem_prop$scr

san_fem_prop$Temperature <- factor(san_fem_prop$Temperature, levels = c("Stable", "Heatwave"))

fem_prop<-glmer(cbind(female, male) ~ (Temperature + Pheromone + as.numeric(gen))^3 + (1|line), data = san_fem_prop, family = binomial,control = glmerControl(optimizer ='bobyqa', optCtrl=list(maxfun=100000)))

drop1(fem_prop, test = "Chi")

fem_prop<-glmer(cbind(female, male) ~ (Temperature + Pheromone + as.numeric(gen))^2 + (1|line), data = san_fem_prop, family = binomial,control = glmerControl(optimizer ='bobyqa', optCtrl=list(maxfun=100000)))

drop1(fem_prop, test = "Chi")

fem_prop <- update(fem_prop,~.-Pheromone:as.numeric(gen))

drop1(fem_prop, test = "Chi")

fem_prop <- update(fem_prop,~.-Temperature:Pheromone)

drop1(fem_prop, test = "Chi")

fem_prop<-glmer(cbind(female, male) ~ Temperature + Pheromone + as.numeric(gen) + (1|line), data = san_fem_prop, family = binomial,control = glmerControl(optimizer ='bobyqa', optCtrl=list(maxfun=100000)))

drop1(fem_prop, test = "Chi")

overdisp_fun(fem_prop) #1.47

summary(fem_prop)

#diagnostic plots

simulateResiduals(fittedModel = fem_prop, plot = TRUE)

femprop_dummy <- data.frame(

gen = rep(0:7, times = 4), # 8 generations

Temperature = rep(c("Stable", "Heatwave"), each = 16),

Pheromone = rep(c("Control", "Treatment"), each = 8, times = 2)

)

predicted_femprop <- predict(

fem_prop,

newdata = femprop_dummy,

re.form = NA, # Exclude random effects

type = "response",

se.fit = TRUE

)

# Create predicted values dataframe

predicted_values_femprop <- data.frame(

femprop_dummy,

predict = predicted_femprop$fit,

se = predicted_femprop$se.fit

)

# Add confidence intervals

predicted_values_femprop$lowerCI <- predicted_values_femprop$predict - 1.96 * predicted_values_femprop$se

predicted_values_femprop$upperCI <- predicted_values_femprop$predict + 1.96 * predicted_values_femprop$se

predicted_values_femprop$Temperature <- factor(predicted_values_femprop$Temperature, levels = c("Stable", "Heatwave"))

fig_6a <- ggplot(san_fem_prop, # Replace with your actual data frame

aes(

x = as.numeric(gen),

y = female / (male + female),

shape = Pheromone,

linetype = Pheromone,

colour = Pheromone,

fill = Pheromone

)) +

geom_point(size = 2.2, alpha = 0.3, position = position_dodge(width =0.40)) +

scale_x_continuous(breaks = 0:7) +

# Add ribbon for confidence intervals

geom_ribbon(data = predicted_values_femprop,

aes(x = gen, ymin = lowerCI, ymax = upperCI, fill = Pheromone),

alpha = 0.4, inherit.aes = FALSE) +

# Add line for predicted values

geom_line(data = predicted_values_femprop,

aes(x = gen, y = predict, colour = Pheromone), linewidth = 1) +

scale_color_manual(values = c("#1b9e77", "#d95f02"),

name = "Pheromone",

labels = c("Pheromone control", "Pheromone treatment")) +

scale_fill_manual(values = c("#1b9e77", "#d95f02"),

name = "Pheromone",

labels = c("Pheromone control", "Pheromone treatment")) +

scale_shape_manual(values = c(17, 16),

name = "Pheromone",

labels = c("Pheromone control", "Pheromone treatment")) +

scale_linetype_manual(values = c("solid", "dashed"),

name = "Pheromone",

labels = c("Pheromone control", "Pheromone treatment")) +

facet_grid(~Temperature) +

labs(

x = "Generation",

y = "Proportion of females"

) +

theme_light() +

theme(

panel.grid.minor = element_blank(),

legend.key.size = unit(1, 'cm'),

legend.key.height = unit(1, 'cm'),

legend.key.width = unit(1, 'cm'),

legend.title = element_text(size=14, face = "bold"),

legend.text = element_text(size=14),

legend.position = "bottom",

axis.text = element_text(size = 14),

axis.title = element_text(size = 14, face = "bold"),

strip.text.x = element_text(size = 14, color = "black"),

strip.text.y = element_text(size = 14, color = "black"),

strip.background = element_rect(

color = "black",

fill = "#F2F4B5",

size = 1.5,

linetype = "solid"

)

)

# Display the graph

fig_6a

# female second count ------------------------------------------------------

san_fem_first = subset(san_morph, count== "Second adult count" & total != 0 & gen != 8)

hist(san_fem_first$female)

san_fem_first$Temperature <- factor(san_fem_first$Temperature, levels = c("Stable", "Heatwave"))

fem_count1<-lmer(female ~ (Temperature + Pheromone + as.numeric(gen))^3 + (1|line), data = san_fem_first)

drop1(fem_count1, test = "Chi")

fem_count1<-lmer(female ~ (Temperature + Pheromone + as.numeric(gen))^2 - Temperature:as.numeric(gen)+ (1|line), data = san_fem_first)

drop1(fem_count1, test = "Chi")

fem_count1<-lmer(female ~ (Temperature + Pheromone + as.numeric(gen))^2 - Temperature:as.numeric(gen) - Temperature:Pheromone + (1|line), data = san_fem_first)

drop1(fem_count1, test = "Chi")

#diagnostic plots

simulateResiduals(fittedModel = fem_count1, plot = TRUE)

summary(fem_count1)

anova(fem_count1)

fem_dummy <- data.frame(

gen = rep(0:7, times = 4), # 8 generations

Temperature = rep(c("Stable", "Heatwave"), each = 16),

#count = rep(c("First adult count", "Second adult count"), each = 16, times = ),

Pheromone = rep(c("Control", "Treatment"), each = 8, times = 2)

)

predicted_fem <- predict(

fem_count1,

newdata = fem_dummy,

re.form = NA, # Exclude random effects

type = "response",

se.fit = TRUE

)

# Create predicted values dataframe

predicted_values_fem <- data.frame(

fem_dummy,

predict = predicted_fem$fit,

se = predicted_fem$se.fit

)

# Add confidence intervals

predicted_values_fem$lowerCI <- predicted_values_fem$predict - 1.96 * predicted_values_fem$se

predicted_values_fem$upperCI <- predicted_values_fem$predict + 1.96 * predicted_values_fem$se

predicted_values_fem$Temperature <- factor(predicted_values_fem$Temperature, levels = c("Stable", "Heatwave"))

fig_6b <- ggplot(san_fem_first,

aes(

x = as.numeric(gen),

y = female,

shape = Pheromone, # Different shapes for points

linetype = Pheromone, # Different line types for trends

colour = Pheromone,

fill = Pheromone

)) +

geom_point(size = 2.2, alpha = 0.3, position = position_dodge(width =0.40)) +

scale_x_continuous(breaks = 0:7) +

scale_color_manual(values = c("#1b9e77", "#d95f02"),

name = "Pheromone",

labels = c("Pheromone control", "Pheromone treatment")) +

scale_fill_manual(values = c("#1b9e77", "#d95f02"),

name = "Pheromone",

labels = c("Pheromone control", "Pheromone treatment")) +

scale_shape_manual(values = c(17, 16),

name = "Pheromone",

labels = c("Pheromone control", "Pheromone treatment")) +

scale_linetype_manual(values = c("solid", "dashed"),

name = "Pheromone",

labels = c("Pheromone control", "Pheromone treatment")) +

# Add ribbon for confidence intervals

geom_ribbon(data = predicted_values_fem,

aes(x = gen, ymin = lowerCI, ymax = upperCI, fill = Pheromone),

alpha = 0.4, inherit.aes = FALSE) +

# Add line for predicted values

geom_line(data = predicted_values_fem,

aes(x = gen, y = predict, colour = Pheromone, linetype = Pheromone), linewidth = 1) +

facet_grid(~Temperature) +

labs(

x = "Generation",

y = "Number of females"

)+

theme_light() +

theme(

panel.grid.minor = element_blank(),

legend.key.size = unit(1, 'cm'),

legend.key.height = unit(1, 'cm'),

legend.key.width = unit(1, 'cm'),

legend.title = element_text(size=12, face = "bold"),

legend.text = element_text(size=12),

legend.position = "bottom",

axis.text = element_text(size = 14),

axis.title = element_text(size = 14, face = "bold"),

strip.text.x = element_text(size = 13, color = "black"),

strip.text.y = element_text(size = 13, color = "black"),

strip.background = element_rect(

color = "black",

fill = "#F2F4B5",

size = 1.5,

linetype = "solid"

)

)

# Display the graph

fig_6b

fig_6<-ggarrange(

fig_6a,

fig_6b,

nrow = 2,

labels = c('a', 'b'),

common.legend = TRUE,

legend = "bottom",

font.label = list(size = 20, face = "bold")

)

ggsave(

filename = "fig_6.pdf",

plot = fig_6,

dpi = 900

)
